# Models of effectiveness of interventions against malaria transmitted by *Anopheles albimanus*

**DOI:** 10.1186/s12936-019-2899-3

**Published:** 2019-08-01

**Authors:** Olivier J. T. Briët, Daniel E. Impoinvil, Nakul Chitnis, Emilie Pothin, Jean Frantz Lemoine, Joseph Frederic, Thomas A. Smith

**Affiliations:** 1 Department of Epidemiology and Public Health, Swiss Tropical and Public Health Institute, 4051 Basel, Switzerland; 2 University of Basel, Petersplatz 1, Basel, Switzerland; 3 Centers for Disease Control and Prevention, Division of Parasitic Diseases and Malaria/Entomology Branch, 1600 Clifton Road, Mail Stop-G49, Atlanta, GA 30329 USA; 4 Programme National de Contrôle de la Malaria, Port-au-Prince, Haiti

**Keywords:** Malaria, *Anopheles*, Haiti, Elimination, *Plasmodium falciparum*

## Abstract

**Background:**

Most impact prediction of malaria vector control interventions has been based on African vectors. *Anopheles albimanus*, the main vector in Central America and the Caribbean, has higher intrinsic mortality, is more zoophilic and less likely to rest indoors. Therefore, relative impact among interventions may be different. Prioritizing interventions, in particular for eliminating *Plasmodium falciparum* from Haiti, should consider local vector characteristics.

**Methods:**

Field bionomics data of *An. albimanus* from Hispaniola and intervention effect data from southern Mexico were used to parameterize mathematical malaria models. Indoor residual spraying (IRS), insecticide-treated nets (ITNs), and house-screening were analysed by inferring their impact on the vectorial capacity in a difference-equation model. Impact of larval source management (LSM) was assumed linear with coverage. Case management, mass drug administration and vaccination were evaluated by estimating their effects on transmission in a susceptible-infected-susceptible model. Analogous analyses were done for *Anopheles gambiae* parameterized with data from Tanzania, Benin and Nigeria.

**Results:**

While LSM was equally effective against both vectors, impact of ITNs on transmission by *An. albimanus* was much lower than for *An. gambiae*. Assuming that people are outside until bedtime, this was similar for the impact of IRS with dichlorodiphenyltrichloroethane (DDT) or bendiocarb, and impact of IRS was less than that of ITNs. However, assuming people go inside when biting starts, IRS had more impact on *An. albimanus* than ITNs. While house-screening had less impact than ITNs or IRS on *An. gambiae*, it had more impact on *An. albimanus* than ITNs or IRS. The impacts of chemoprevention and chemotherapy were comparable in magnitude to those of strategies against *An. albimanus*. Chemo-prevention impact increased steeply as coverage approached 100%, whilst clinical-case management impact saturated because of remaining asymptomatic infections.

**Conclusions:**

House-screening and repellent IRS are potentially highly effective against *An. albimanus* if people are indoors during the evening. This is consistent with historical impacts of IRS with DDT, which can be largely attributed to excito-repellency. It also supports the idea that housing improvements have played a critical role in malaria control in North America. For elimination planning, impact estimates need to be combined with feasibility and cost-analysis.

**Electronic supplementary material:**

The online version of this article (10.1186/s12936-019-2899-3) contains supplementary material, which is available to authorized users.

## Background

*Anopheles albimanus* is the main vector in malaria foci in Central America and the Caribbean, in particular in Haiti [1, 2], which accounts for most of the *Plasmodium falciparum* transmission in the Caribbean. *Anopheles albimanus* has very different characteristics from those of the *Anopheles gambiae* species complex and *Anopheles funestus* group, which comprise the main malaria vectors in Africa, and which have been most intensively studied. *Anopheles albimanus* has a higher intrinsic mortality rate (Additional file 1), is much more zoophilic and is much less likely to rest in houses [1, 3]. Also, importantly, it has a tendency towards crepuscular biting, although this may vary extensively depending on the location [1, 3]. Due to these differences, the impacts of different interventions on malaria transmitted by *An. albimanus* are likely to differ substantially from those estimated from trials in Africa, and the ranking of vector control interventions in terms of effectiveness may also be different. Therefore, the prioritization of vector control interventions in Central America and the Caribbean, in particular for eliminating *P. falciparum* from Haiti, should take local vector bionomic characteristics into account.

Malaria transmission in Central America and the Caribbean is low, with 4.6 reported cases (presumed and confirmed) per 1000 population at risk, compared to Africa with 189.6 cases per 1000 [4]. Effects of naturally acquired immunity on transmission are therefore likely to be negligible, and the effect of vector control interventions on transmission may be examined in terms of the effect on the vectorial capacity. Similarly, the effect of interventions against malaria infections in humans, such as chemo-prevention, might be studied in terms of the effects on the reproduction number in simple susceptible-infected-susceptible (SIS) models.

This study used data from field observations in Haiti and data extracted from published literature to parameterize mathematical models of malaria transmitted by *An. albimanus*, and the impact of interventions against it. As well, impacts were compared to that of these interventions against *An. gambiae*. The results are presented in terms of the effect of varying the coverage of different interventions.

## Methods

### Modelling framework

In accordance with the original purpose of the concept of vectorial capacity [5], vector control effects were summarized by computing the impact of interventions on this quantity, using a discrete time entomological model of the oviposition cycle [6]. Malaria is modelled using the SIS model of [7], which can be described by:

$$I_{t + 1} = \frac{\beta }{N}I_{t} \left( {N - I_{t} } \right) +{\upgamma}I_{t}$$

where $I_{t}$ is the number of infectious individuals at time *t*; *N*, the total human population; $\beta$, the contact rate (the number of individuals with whom an infectious individual makes enough contact to pass infection in one time-step); and $\gamma$, the proportion of the infectious population that remains infectious at next time step. The value of the basic reproduction number is the product of the contact rate, *β*, and the average duration of the infectious period, $1/\left( {1 -\upgamma} \right)$,

$$R_{0} = \frac{\beta }{1 - \gamma }.$$

Effects of interventions on vectors were captured by an existing model for the dynamics of malaria in a mosquito population feeding on, infecting and getting infected from a heterogeneous population of hosts [6], which provides values of the vectorial capacity, which is equal to $\beta$. This model represents a population of female host-seeking mosquitoes with a system of difference equations for the total number of vectors and the number of infected and infectious vectors, with probabilities for surviving through each stage of the gonotrophic cycle. Competition between different types of hosts, including animals, is included, and mosquitoes that fail to feed on any night are assumed to return to host-seeking state each night until they either obtain a blood meal or die. A mosquito’s behaviour in any gonotrophic cycle is assumed to be independent of its behaviour in previous cycles.

The main differences between this framework and the classic Ross-Macdonald model [8, 9] are the explicit modelling of different stages of the oviposition cycle, linked to a fixed resting period, and relatedly, the use of difference equations rather than ordinary differential equations in continuous time.

### Parameterization of models in the absence of interventions

The review of Sinka and colleagues [1] was used as the primary source for literature on relevant bionomics of *An. albimanus.* Additional information was extracted from more recent publications [2, 10, 11]. Published values were sought for the human blood index (HBI), the biting rhythm, mortality or survival (as measured by the parous rate), and the duration of the resting period. Rather than using a formal meta-analysis, relevant values for Haiti were chosen based on the data sources listed in Table 1. Other bionomics data is listed in the supplementary information (Additional file 1).

**Table 1:** Bionomic parameter values of *An. albimanus* in Hispaniola extracted from the literature, and *An. gambiae* in Tanzania

Parameter	Symbol	*An. albimanus*	*An. gambiae*
Human blood index	χ	5.4% [41]^a^	0.939 (see Additional file 2)
Biting rhythm		Table in Additional file 1 [Impoinvil, unpublished data]	Pattern in Ulanga reported by Huho et al. [17]. See Fig. 2 and Additional file 4
Parous rate	$M$	0.484 [42]^b^	0.623 (see Additional file 2)
Resting period duration	$\tau$	3.5 days [42]^c^	3 days (see Additional file 2)
Sac rate^d^	$A_{0}$	0.405 [42]	0.313 (see Additional file 2)

^a^The human blood positivity rates (HBPR) for indoor (n: 1232), peridomestic (n: 466) and corral (n: 136) samples of *An. albimanus* were 13.3, 5.4 and 4.4%, respectively. The weighted average is 10.6%, but this is biased because most mosquitoes came from indoor collections. The unweighted average is 7.7%

^b^Bellevue site

^c^Time required for a mosquito that has encountered a host to return to host-seeking, provided that the mosquito survives to search again. Molez et al. [43] found a mean for 3.5 days in parous females and 5.4 days in nulliparous females. In the model, which requires an integer value, 4 days was used

^d^The proportion of mosquito ovaries with sacs, used to indicate that oviposition occurred on the previous day, and hence used to estimate the duration of the oviposition interval

This model was implemented with daily time-steps and γ = 0.995 corresponding to exponential survival of untreated infections with an average duration of 200 days [12, 13]. Supplemental input parameters required for the entomological model (Additional file 2) are given in Table 2. The values of $\tau_{d}$, $P_{B}$, $P_{C}$, $P_{D}$ for non-intervened hosts are those used in previous analyses of transmission by *An. gambiae* sensu stricto (*s.s*.) [6].

**Table 2:** Standard parameters of the entomological model without intervention (see Additional file 2)

Parameter	Symbol	Value
Maximum length of time that a mosquito searches for a host in one 24 h period if it is unsuccessful	$\tau_{d}$	0.33 days
Probability that a mosquito bites after encountering a host	$P_{B}$	0.95
Probability that a mosquito finds a resting place after biting	$P_{C}$	0.95
Probability that a mosquito survives the resting phase after biting	$P_{D}$	0.46
Probability that a mosquito lays eggs and returns to host-seeking after biting	$P_{E}$	0.88

Field data on the biting rhythm of *An. albimanus* were obtained in a recently conducted study in Haiti (Impoinvil, personal communication) and extracted from literature and summarized in Additional file 1. The rhythm found in the Dame Marie site, with peaks at 8:30 PM and 4:30 AM and was used for the main analysis. A rhythm with extremely early biting (peak at 6:15 PM), from data pooled over three locations in northern and central Haiti in a study by Taylor [14], and a rhythm with a biting peak at 1.30 AM from a study in Laborde by Desenfant [15], were also used to determine the sensitivity of the analysis to this parameter.

These biting rhythms were aligned with data on the human activity rhythm from a survey conducted in a Haitian community without electricity [16] to adjust for the early-evening and early morning biting in the model of ITNs. The corresponding analysis for *An. gambiae* used values from Ulanga in Tanzania [17]. These data were used to estimate the proportion of human exposure to mosquito bites of a given vector population which occurs while the host is in bed in the absence of any protective measure (*π*_*i*_) [18].

#### Zoophily

The model calculates the relative availability of human and non-human blood to mosquitoes based on the HBI and the proportion of the number of humans out of the total number of potential hosts (set to 50%).

#### Survival

The field estimate of the parous rate, $M$, provides an indirect estimate of the survival rate of an *Anopheles* mosquito [19] per gonotrophic cycle. This is much lower for *An. albimanus* than for *An. gambiae*.

#### Duration of the extrinsic cycle

The mean duration of the extrinsic cycle of the parasite was obtained by applying Moshkovsky’s formula [20] using the average temperature for Port-au-Prince (Haiti) of 28.1 °C. This gave values of $\theta_{s} = 9.2$ days for *P. falciparum* and $\theta_{s} = 7.7$ days for *P. vivax*.

### Modelling and parameterisation of intervention effects

The effects on transmission of interventions, applied either to humans or vectors can be captured as reductions in $\beta$ to $\beta '$, or decrease in $\gamma$ to $\gamma '$, so that in general, the reproduction number under control is:

$$R_{c} = \frac{\beta '}{1 - \gamma '},$$

and the effectiveness of an intervention in reducing transmission is measured by:

$$1 - \frac{{R_{c} }}{{R_{0} }} = 1 - \frac{{\left( {1 - \gamma } \right)\beta '}}{{\left( {1 - \gamma '} \right)\beta }}$$

Combinations of interventions can be evaluated by assuming independent effects on $R_{c}$, which is a reasonable approximation for most, but not all, intervention combinations.

#### ITNs and IRS

Entomological interventions do not affect $\gamma$. Their effects on the vectorial capacity, and hence on $\beta$ were obtained by running the entomological model. For each intervention its repellent effect (preventing mosquitoes from attacking a human) was modelled as a reduction in the availability of humans, to be bitten by mosquitoes $\alpha_{1}$. The reduction was calculated as the proportion *π*_*i*_ multiplied by the repellent effect as measured in field studies. Similarly, intervention effects on mosquito survival before and after taking a blood meal were modelled by adjusting values of $P_{B}$ and $P_{C}$, respectively, for each intervention. The parameterization of these intervention effects, based on field data on *An. albimanus* from Southern Mexico and Belize and on *An. gambiae* from Benin and north-west Nigeria, is detailed in the supplementary information (Additional file 3). No resistance to the insecticides used for these interventions has been detected in Haiti.

#### House-screening and spatial repellents

Indoor deployment of a transfluthrin spatial repellent in Belize has been found to reduce house entry of *An. albimanus* by 56% [11]. This is equivalent to the expected reduction in availability of humans, and is very similar to the reduction of 59% anticipated with house-screening [21], so the same model was used for spatial repellents and for house screening (with an assumed reduction of 59%).

#### Larval source management

The impact on vectorial capacity in this model is identical to the proportionate reduction in emergence of female mosquitoes. Assuming that the effect of LSM is to eliminate or make whole breeding sites unproductive, the coverage was defined as the proportion of breeding sites treated. The proportionate reduction of the vectorial capacity achieved by LSM is thus equal to the coverage.

#### Vaccination

The RTS,S malaria vaccine recently demonstrated partial efficacy in a large paediatric Phase III trial [22]. A mass vaccination program with such a vaccine was modelled, assuming the same efficacy and persistence of protection in all ages of hosts, and that the specified coverage corresponds to the proportion of the population vaccinated in any two year period (equivalent to a rolling programme at steady state). Allowing for the dynamics of efficacy over time estimated for 5–17 month old children in the Phase III trials (with a Weibull decay model), and assuming a 3-month period with no efficacy, corresponding to the initial period of vaccination [23] the mean efficacy over the two-year period is 37%, so that in the SIS model, the effect of a vaccination programme with coverage $\omega$, is to multiply the transmission parameter, $\beta$, so that $\beta^{\prime} = \left( {1 - 0.37\omega } \right)\beta$. Vaccination is assumed to occur via a rolling programme that operates throughout the year so seasonality in transmission does not need to be considered.

#### Treatment of symptomatic falciparum malaria

The effect of treatment on transmission of *P. falciparum* was modelled using the simplification that symptomatic episodes of malaria are most likely to occur at the start of the infection, and can thus be incorporated into the SIS transmission model as a reduction in the force of infection by the proportion ($\upalpha$) of new infections that are effectively treated. When a new symptomatic super-infection is treated, pre-existing asymptomatic infections are also cleared, so that the extended SIS model is:

$$I_{t + 1} = \frac{{\beta \left( {1 -\upalpha} \right)}}{N}I_{t} \left( {N - I_{t} } \right) + \gamma \left( {1 - \frac{{\beta\upalpha}}{N}I_{t} } \right)I_{t}$$

with:

$$R_{c} = \frac{{\beta \left( {1 -\upalpha} \right)}}{1 - \gamma }.$$

**Fig. 1:**
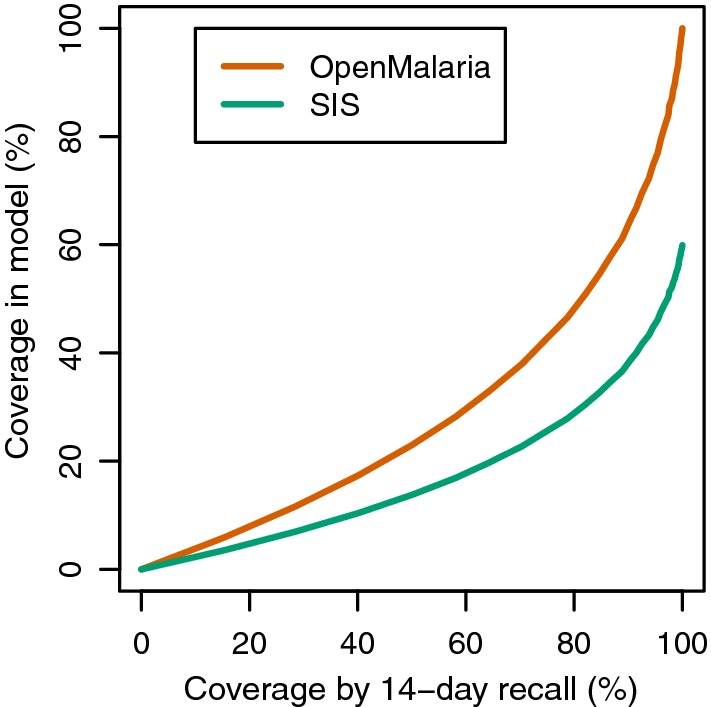
Calibration of model parameters measuring treatment coverage against reported coverage in 14-day recalls

Standard survey data use 14-day recalls to quantify access to effective case management. A previous exercise to calibrate OpenMalaria simulation models against such surveys [24] was extended by computing the ratio of treatments to new infections in simulations of low transmission settings in OpenMalaria, thus providing a mapping of the proportion of new infections treated corresponding to any value of coverage based on 14-day recalls (Fig. 1).

The same model of impact can be used to capture effects of surveillance-response systems that depend on searching for clinical cases.

#### Treatment of asymptomatic falciparum infections

A simple model that approximates various interventions involving treatment of infections in the community, irrespective of clinical status (such as test and treat strategies), is

$$I_{t + 1} = \varepsilon \left( {\frac{{\beta I_{t} }}{N}\left( {N - I_{t} } \right) + \gamma I_{t} } \right),$$

where $\varepsilon$ is the proportion of infections that escape treatment at any deployment of the intervention.

Assuming independence between intervention deployments, and $\delta$ is the length of time between deployments expressed in years, the annual coverage, $\upalpha$, i.e. the proportion of the population that receives the intervention each year, is

$$\upalpha = 1 -\upvarepsilon^{1/\delta } ,$$

and the controlled reproduction number is

$$R_{c} = \frac{\varepsilon \beta }{1 - \varepsilon \gamma } = \frac{\beta }{1/\varepsilon - \gamma } = \frac{\beta }{{\left( {1 - \alpha } \right)^{ - \delta } - \gamma }}.$$

So the effectiveness in reducing $R_{c}$ is

$$1 - \frac{{\left( {1 - \gamma } \right)}}{{\left( {1 - \alpha } \right)^{ - \delta } - \gamma }}.$$

#### *Plasmodium malariae* and *Plasmodium vivax*

*Plasmodium malariae* also occurs in Haiti, but is very infrequent [25] and it is unclear whether *P. vivax* occurs [26]. The models for vector-control can be applied to either of these species after adjustment of the duration of the extrinsic incubation period (Table 1). Models for the effects of human-side interventions on *P. vivax* and *P. malariae* are outside the scope of this paper.

#### Intervention combinations

The analysis did not explicitly consider combinations of interventions.

## Results

**Fig. 2:**
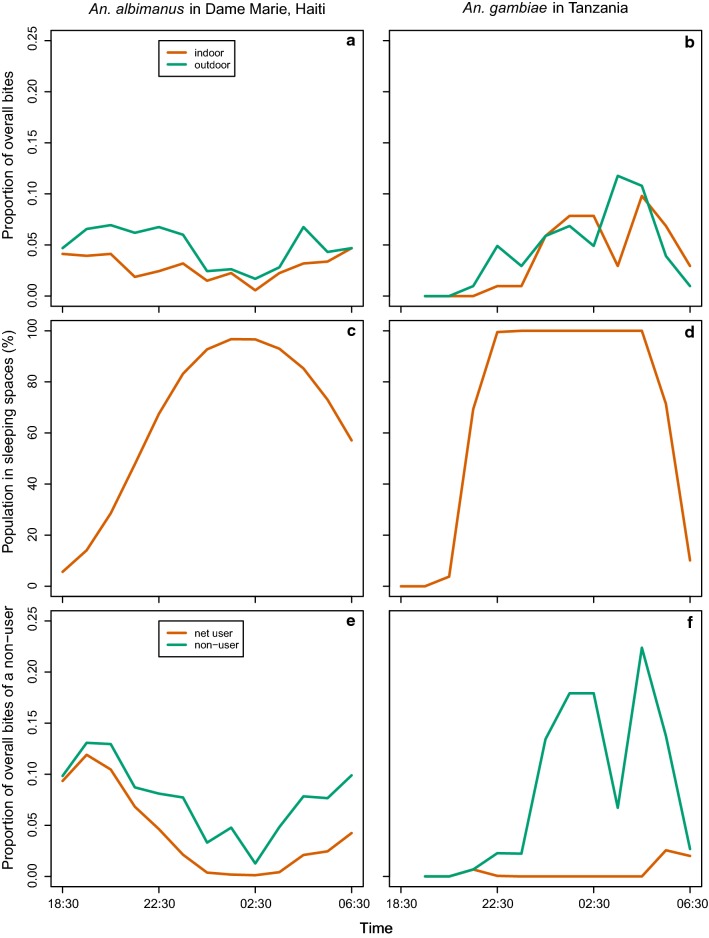
Alignment of activity rhythms of mosquitoes and humans. **a** Biting rhythms from human landing catches of *An. albimanus* at Dame Marie; **b** Biting rhythms from human landing catches of *An. gambiae s.l.* [17]; **c** Human activity rhythm in Haiti [16]; d Human activity rhythm in Tanzania [44]; e and f Exposure to mosquito bites for net users (assuming nets avert 100% of exposure) and nonusers, assuming that people are outside when not in bed, by hour

The biting rhythm of *An. albimanus*, derived from the human landing catch data recently collected in Dame Marie (Fig. 2a) indicates that most of the exposure to bites by this species is around dusk and dawn. The data used for the human activity rhythm (Fig. 2c) indicate substantial variability in the time that people are in bed. When these data are combined with the *An. albimanus* biting rhythm data to calculate exposure by hour (Fig. 2e), most of the exposure is seen to occur in the early evening and late morning, irrespective of whether bed nets are used. Under the assumption that people are outdoors before bedtime (and after waking up) the proportion of mosquito bites that would be prevented by ITNs (*π*_*i*_) for the Dame Marie location was 0.49, while for the extreme early biting rhythm observed by Taylor [14] in northern and central Haiti (see Fig. 1 in Additional file 1), the *π*_*i*_ was only 0.08. In the Laborde site, where Desenfant [15] observed the biting peak to occur at the time when most people are asleep (see Fig. 1 in Additional File 1), the *π*_*i*_ was 0.75, still below to the *π*_*i*_ of 0.95 observed for *An gambiae* in Tanzania (Fig. 2f), which bites during the middle of the night when most people are in bed (Fig. 2b and d). With the alternative assumption that people are indoors when not in bed, the *π*_*i*_ value was 0.57 for the Dame Marie location, 0.23 for northern and central Haiti and 0.79 in Laborde, while being 0.90 for Tanzania.

**Fig. 3:**
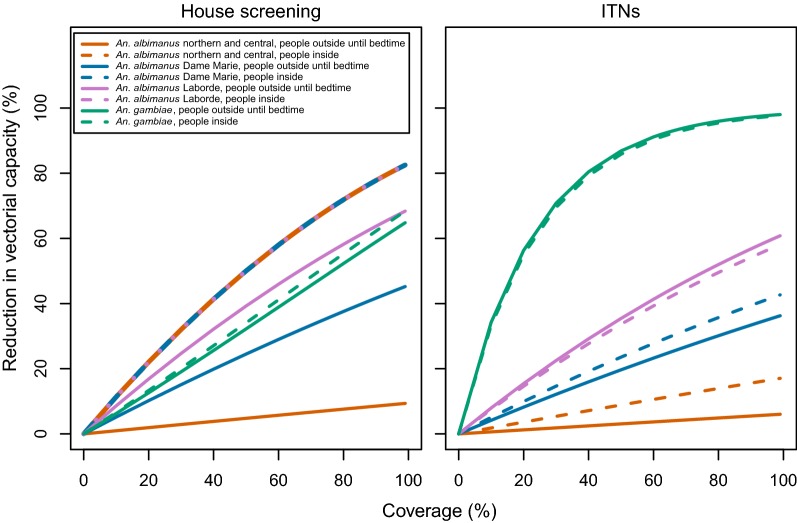
Intervention impact based on coverageof house screening and bed nets on transmission by *An. albimanus* from various locations and *An. gambiae.* Left panel: house screening; right panel: ITNs

**Fig. 4:**
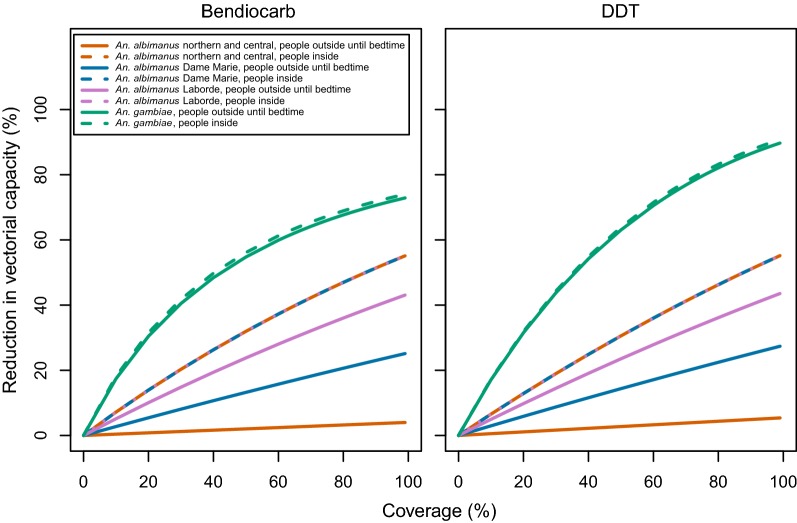
Impact of IRS on transmission depending on the vector and location of people in the evening, as well as coverage. Left panel: bendiocarb; right panel: DDT

**Fig. 5:**
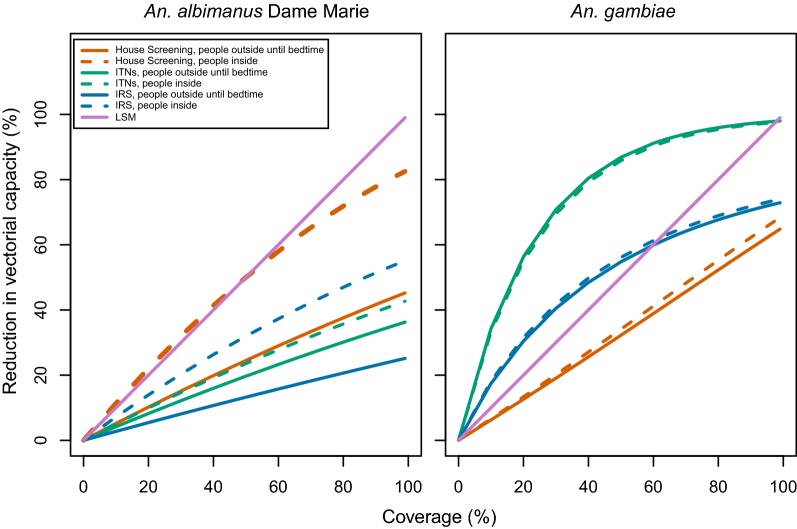
Impact of vector control on transmission by *An. albimanus* in Dame Marie and *An. gambiae.* IRS active ingredient is bendiocarb. Left panel: *An. albimanus* in Dame Marie; right panel: *An. gambiae*

Due to these and other differences in the bionomics of *An. albimanus* and *An. gambiae*, the predicted impact of ITNs on the vectorial capacity was low in Haiti compared to Tanzania (Fig. 3 and Table 3), irrespective of the assumption about where people are when not in bed. If it was assumed that people are outdoors until they go to bed, a similar impact was predicted for IRS with DDT or bendiocarb as for ITNs (Figs. 4, 5 and Table 3). However, if it was assumed that people are indoors by the time that *An. albimanus* commences biting, the impact of IRS was much stronger than that of ITNs. The impact of each of these vector control interventions against *An. albimanus* was close to linear in coverage (Fig. 5), while for *An gambiae* more curvature was present. The vector control intervention having the highest impact on *An. albimanus* at a given coverage was LSM. The impact of LSM on the vectorial capacity was numerically equal to the coverage, as measured by the proportionate reduction in the emergence rate. House-screening, and hence spatial repellents, attained similar effects to LSM at the same coverage under the assumption that people are indoors by the time that *An. albimanus* bites, but the effect of house screening showed some saturation at high coverages.

**Table 3:** Percentage of reduction of vectorial capacity and reproduction at 10% coverage with vector control or drug related interventions

Intervention	Active ingredient	Evening location of people (until bed time)	*An. albimanus* northern and central Haiti	*An. albimanus* Dame Marie	*An albimanus* Laborde	*An. gambiae*
IRS	Bendiocarb	Inside	7.21	7.17	7.17	18.15
		Outside	0.40	2.74	5.13	17.41
	DDT	Inside	6.55	6.55	6.55	17.27
		Outside	0.55	2.96	4.95	17.08
	δ	Inside	2.44	2.49	2.49	22.38^a^
		Outside	0.04	0.66	1.55	21.27^a^
	λ	Inside	NA	NA	NA	9.77
		Outside	NA	NA	NA	9.36
ITNs	λ	Inside	0.62	4.17	7.94	34.24
		Outside	1.82	5.06	7.51	33.30
House screening	NA	Inside	11.35	11.35	11.35	6.60
		Outside	0.97	5.17	8.61	6.25
LSM	NA	NA	10.0	10.0	10.0	10.0
RTS,S vaccination	NA	NA	3.70	3.70	3.70	3.70
Test and treat	NA	NA	5.47	5.47	5.47	5.47
Case management	NA	NA	2.30	2.30	2.30	2.30

*IRS* indoor residual spray, *ITNs* insecticide treated nets, *LSM* larval source management, *δ* deltamethrin, *λ* lambdacyhalothrin, *NA* not applicable/available

**Fig. 6:**
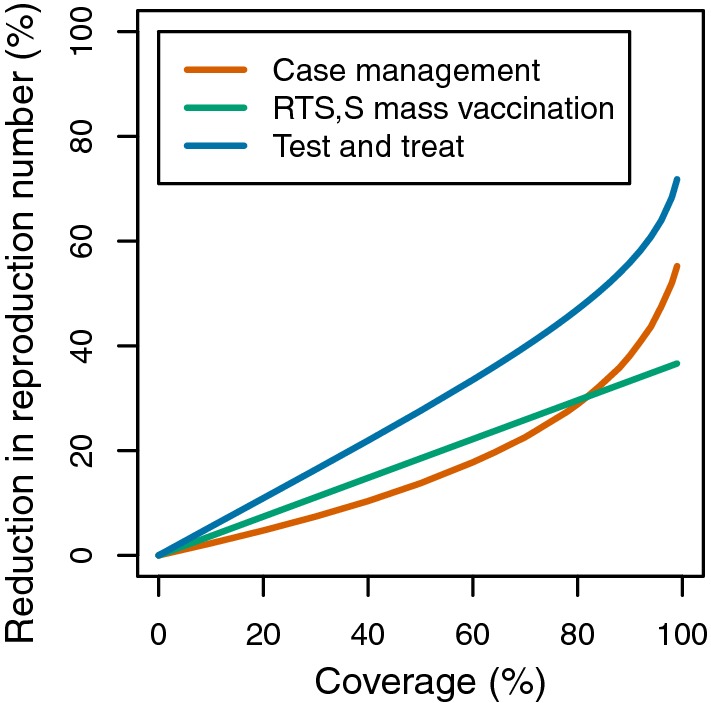
Vaccine and drug intervention impact on transmission by coverage

^a^Parameterized using data from studies with *An. albimanus*. The impact of vector control is on vectorial capacity, and the impact of human side interventions is on the reproduction number. The impact of higher coverage is approximately proportional to the coverage for most interventions over most of the range (see Figs. 3, 4, 5, 6)

At equal coverage, RTS,S mass vaccination (Fig. 6) was predicted to have a lower impact than ITNs (except with the very early biting rhythm observed in northern and central Haiti by Taylor [14]), but the impact relative to IRS depended on the insecticide, the biting rhythm, and whether people are indoors or outdoors when not in bed (Table 3). The impacts of chemoprevention and chemotherapy were predicted to be non-linear in coverage (Fig. 6). A key determinant of the effectiveness of chemo-prevention strategies is the proportion of the population that is missed [27], so effectiveness increased steeply as coverage approaches 100%. For case management, the slope of the coverage-effectiveness curve also increased with coverage.

**Fig. 7:**
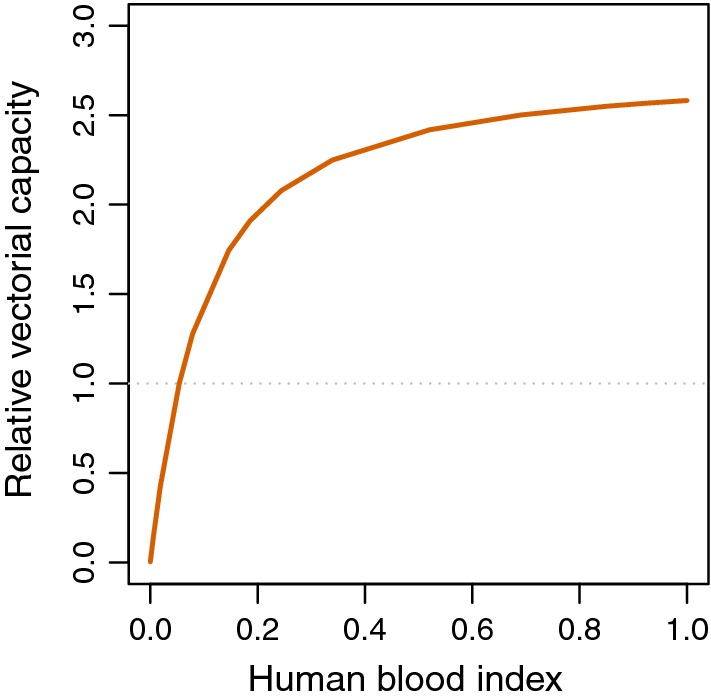
Sensitivity of transmission estimates to human blood index. Relative vectorial capacity in the absence of interventions (relative to HBI = 0.1)

The finding that interventions with deterrent effects were relatively effective in reducing the vectorial capacity suggested that their impact might be sensitive to the availability of alternative hosts. Since the ratio of humans to animals in the local environment is obviously highly variable, so are the potential effects of zoophagy on malaria transmission. In the absence of interventions, vectorial capacity was predicted to initially strongly increase with the proportion of mosquito feeds on humans, but to saturate at high HBI levels (Fig. 7). The implications of this were investigated by analysis of the sensitivity to variations in the HBI of the proportionate reduction in transmission achieved by house screening. However, the percentage impact of house-screening was independent of the HBI.

## Discussion

Calculating impacts on vectorial capacity provides clear general statements about impacts of entomological interventions, without the need to estimate prevailing levels of transmission or infectiousness. This was the original motivation of Garrett-Jones when he devised the vectorial capacity in 1964 [5]. The numerical equivalence of the vectorial capacity with the transmission parameter in the SIS model provides an easy way of comparing likely impacts of vector control with those of human-side interventions, because of the equivalence of effects on vectorial capacity and on the reproduction number (because a reduction in vectorial capacity is proportional to the ensuing reduction in reproduction number).

For *An. albimanus*, the slopes of the curves in Table 3 provide a concise way of comparing the potential impacts of the interventions, since most of them are close to straight lines below 80% coverage. However, these need to be combined with an analysis of operations and costs involved in reaching different levels of coverage if they are to be useful for elimination planning. These will have substantial effects on which intervention mixes are most attractive, since the constraints in achieving a high coverage are different for each intervention. For instance, in this model, the indication that LSM will be the most efficient intervention may be deceptive depending on the scenario. LSM is usually synonymous with larviciding and the World Health Organization recommends larviciding for malaria control in sub-Saharan Africa only in areas where the breeding sites are few, fixed, and findable [28]. In that context, sustained high coverage of larviciding is hard to achieve, entailing thorough searches and recurrent treatment of breeding sites. Indeed, *Anopheles albimanus* in Haiti breeds in diverse types of habitats [1, 2, 10, 29], which means that it is unlikely that breeding sites are small in number, fixed in location, or easy to find. However, in Haiti, where elimination is the goal, it may be possible to conduct a time-limited, cost-effective LSM intervention in a small, well-defined focus of malaria transmission as a supplement to other core interventions. This will contribute to focal suppression of mosquitoes and subsequent interruption of malaria. Potentially, the development of emerging technology such as drones might facilitate the identification and control of vector breeding sites at various geographical and operational scales in a cost-effective manner [30]. Measurement of coverage as the proportionate reduction in emergence is convenient for parameterizing the model, but estimation of this in practice is likely to be very difficult.

From the model, it is implied that house-screening in Haiti is likely to be a very effective intervention wherever there is malaria transmitted by *An. albimanus*. House-screening entails a one-off modification of houses, and coverage is relatively easy to quantify. However, there is little information available in literature on the coverage of house screening in Haiti. The very substantial predicted impact is consistent with diverse evidence from across the world [31], dating back to the original studies of house-screening in Italy [32]. Substantial impacts of house improvement against malaria transmitted by north American *Anopheles* were documented in the USA in the mid-20^th^ century [33]. Poor-quality housing is well-recognized as a factor contributing to the vulnerability of Haiti to natural disasters, and improving houses is generally popular with their inhabitants. Potentially, improved house construction and screened windows and doors could contribute to elimination of malaria from Haiti. However, the model parameterization is highly uncertain because the review by Tusting and colleagues [31] that it is based on, is a summary of studies of diverse vectors and house architectures, and the predictions are sensitive to assumptions about human behaviour. In Tanzania, there is little difference between these extreme assumptions for *An. gambiae* because the times when people are indoors in Tanzania overlap substantially with those when it is biting. For Haiti, the impact predicted for house-screening is highly dependent on the assumption that people are inside whenever mosquitoes are biting. When the alternative is assumed, i.e. that people stay outside when not in bed, the impact is lower, and the degree to which it is reduced depends on the biting rhythm. Local data are needed to assess which assumption is most realistic in any particular setting. It is quite possible that people increase time inside when houses are screened, or that the time spent inside is dependent on the availability of artificial light.

*Anopheles albimanus* contrasts with the dominant African malaria vectors in its greater tendency to bite in the evening or morning and to feed on animals. These differences lead to substantial discrepancies between estimates of the likely impacts of interventions against malaria transmitted by this vector and systematic reviews of intervention trials [34, 35], which are largely based on data from sites with anthropophilic vectors. The estimated impacts of ITNs and IRS against *An. albimanus* were substantially lower than those for *An. gambiae* (Table 3).

The two extreme assumptions about human location (inside or outside the house) when not in bed did not result in very large differences in impact of ITNs, because ITNs were assumed to only have a protective effect when people are in bed. The predicted impact of ITNs against transmission by *An. albimanus* is relatively poor compared with that of a systematic review of trials of ITNs, mostly against African vectors [34]. It is also consistent with the results of a recent case–control study in Haiti [36] which showed little personal protection, but which was not able to measure the community effect.

In contrast to ITNs, the predicted impact of IRS with DDT or bendiocarb was surprisingly large, but only with the assumption that people are inside (when not in bed) whenever mosquitoes are biting. This assumption may be unreasonable. Taylor [14] writes: “Over three quarters of the biting activity takes place between 17:30 and 21:00 h, the hours during which the majority of Haitians living in the provinces are outdoors.” In contrast to ITNs, IRS was assumed to protect against bites indoors when people were not bed. This may be reasonable, given the apparent effectiveness of DDT against *An. albimanus* in the 1960s, in particular in Haiti [2].

The effect of DDT against *An. albimanus* was purely modelled as a deterrent from house entry, without any killing effect, as the study this was based on Bangs [37] who did not detect a difference in mortality between the treatment and control arms, due to high mortality in the controls.

DDT has been shown to have spatial repellent effects on *An. albimanus*, in contrast to other insecticides [38], and these effects account for the greater impact of DDT in the models, supporting the general conclusion that that the most effective interventions against *An. albimanus* are those that divert the vector from humans to other hosts. Roberts and colleagues [39] also assessed that the dominant actions of DDT residues in reducing man-vector contact inside houses were effects of repellency and irritancy, and showed that these effects were larger with the American vector *Anopheles darlingi* than with *An. gambiae*. This is similar to the conclusions of analyses of zoophilic vectors in India [40]. Whereas IRS with DDT is probably not likely to be implemented in Haiti, spatial repellents such as transfluthrin [11] could well be practicable and affordable options. Spatial repellent emanators represent a less permanent solution than house screening, but they can be deployed adaptively in the times and places when the mosquitoes are biting, and even be effective when deployed near people spending evenings outdoors. On the other hand, permanent house improvements may be popular, even where malaria is not perceived to be a major priority. Similarly, when people deploy repellents, or go inside when mosquitoes are biting, they may be responding to nuisance mosquitoes, rather than to *Anopheles*, but equally they may also be protecting themselves against *Aedes aegypti* and the viruses it can transmit [41]. Such considerations make it hard to estimate the effective coverage that might be reached.

## Conclusion

The models suggest that LSM and interventions such as house-screening that deter or repel *An. albimanus* host-seeking in the evening or morning will have relatively high impact compared to interventions such as bed nets that are more suitable against endophilic night-biting vectors. Interventions directed at humans, including mass vaccination and chemo-prevention, have similar impact on transmission compared to vector control at analogous levels of coverage. Optimal intervention packages for any specific locality can be designed by linking these quantitative results based on vector bionomics to local data on human behaviour and the levels of coverage achievable for the different interventions.

Because Haiti is aiming for elimination, malaria control paradigms from the African context may not apply. Rather, malaria interventions should be considered with an understanding that they will be implemented in a time-limited manner until malaria is eliminated from Haiti. While this analysis aims to guide decision-making in selecting interventions, it will be important for Haiti to weigh predicted impacts by programmatic factors of ease of implementation and expected time to elimination. However, translating these results into a practical algorithm for prioritization of interventions also needs to take into account the local feasibility, and availability of different resources.

## Additional files

**Additional file 1.** Bionomics data sources.

**Additional file 2.** Parameterization of the mosquito gonotrophic cycle.

**Additional file 3.** Parameterization of intervention effects.

**Additional file 4.** R code library.

## Data Availability

All data used in this study are available in the tables and supplementary information of this publication. All computer code used is provided in Additional file 4.

## Abbreviations

DDT: dichlorodiphenyltrichloroethane
HBI: human blood index
HBPR: human blood positivity rates
IRS: indoor residual spraying
ITNs: insecticide-treated nets
LSM: larval source management
s.s.: sensu stricto
SIS: susceptible-infected-susceptible
