# Supplementary material for: Models of effectiveness of interventions against malaria transmitted by Anopheles albimanus
Source: Malar J. 2019 Aug 1;18:263. doi: 10.1186/s12936-019-2899-3 (PMC6670173; doi:10.1186/s12936-019-2899-3)
Supplement: Supplementary file 1 — Additional file 1. Bionomics data sources. [file 12936_2019_2899_MOESM1_ESM.docx]

## Additional file 1

**Table S1. Supplementary bionomic parameter values of *An. albimanus* extracted from the literature**

| Parameter | Symbol | Value | Country | Reference |
| --- | --- | --- | --- | --- |
| Human blood index | χ | 4.4% (9 out of 205) | Dominican Republic | (1) |
|  |  | 0%, 11% (Results for two different kinds of traps) | Belize | (2) |
|  |  | 32.3% (Outdoor collections) | Belize | (3) |
| Biting rhythm |  | Table 2 (Combination of data from sites: Port-de-Paix, Gros Morne and Arcahaie) | Haiti | (4) |
|  |  | Table 3 (Four villages: Morne Anglais, Belle Hotesse, La Fond and St. Michel, all located near Cap Hatien) | Haiti | (5) |
|  |  | Table 4 (sites: EM61 (Bellevue), EM48 and Laborde) | Haiti | (6) |
|  |  | Table 5 (sites: Dame Marie, Artibonite and Ouanaminthe) | Haiti | Impoinville (unpublished) |
|  |  | Table 6 (Four villages: all located near Dajabon Town) | Dominican Republic | (7) |
| Indoor resting |  | 33.4%^[[1]](#endnote-1)^ | Haiti | (5) |
|  |  | 56.8%^[[2]](#endnote-2)^ | Haiti | (4) |
|  |  | 10% | Jamaica | (8) |
|  |  | 34.0%^[[3]](#endnote-3)^ | Coastal plains of southern Mexico | (9) |
| Parous rate | $M$ | 0.29 | Mexico | (10) |
|  |  | 0.37 | Dominican Republic | (7) |
| Resting period^[[4]](#endnote-4)^ | $\tau$ | 3.0 days^[[5]](#endnote-5)^ | Colombia | (11) |
|  |  | 2.2 days^[[6]](#endnote-6)^ | Dominican Republic | (12) |

**Table S2. Biting rhythm of *An. albimanus* in Haiti published by Taylor (4)**

| Mean time | Start time | End time | Outside | Inside |
| --- | --- | --- | --- | --- |
| 18:15 | 17:30 | 19:00 | 946 | 261 |
| 20:00 | 19:00 | 21:00 | 309 | 130 |
| 22:00 | 21:00 | 23:00 | 214 | 69 |
| 00:00 | 23:00 | 01:00 | 158 | 21 |
| 04:00 | 01:00 | 07:00 | 48 | 15 |

**Table S3. Biting rhythm of *An. albimanus* in Haiti published by Hobbs and colleagues (5).**

Means for all-night catches at four villages in northern Haiti. Total catch : 513, mean per night = 51. The biting captures were performed from sundown to sunrise in at least four houses of each study village, on a bimonthly basis.

| Mean time | Start time | End time | Outside | Inside |
| --- | --- | --- | --- | --- |
| 20:00 | 17:10-19:30 | 22:00 | 32.0 | 21.4 |
| 00:00 | 22:00 | 02:00 | 21.6 | 11.1 |
| 03:30 | 02:00 | 05:00 | 10.0 | 2.8 |
| 06:00 | 05:00 | 05:51-06:34 | 3.2 | 1.5 |

**Table S4. Biting rhythm of *An. albimanus* in Haiti published by Desenfant (6).**

Note that these are the same data as published by Molez and colleagues (Molez, Desenfant, & Jacques 1998), with the Bellevue location corresponding to EM 61 provides detailed rhythms from the same places (EM 61 is Bellevue)

| Mean time | Start time | End time | EM61 Outside | EM61 Inside | EM48 Outside | EM48 Inside | Laborde Outside | Laborde Inside |
| --- | --- | --- | --- | --- | --- | --- | --- | --- |
| 17:30 | 17:00 | 18:00 | 1.8 | 0.2 | 0.2 | 0.4 | 0.3 | 0.5 |
| 18:30 | 18:00 | 19:00 | 2.6 | 2.0 | 1.1 | 0.8 | 0.7 | 0.5 |
| 19:30 | 19:00 | 20:00 | 4.0 | 2.1 | 2.3 | 1.6 | 0.5 | 0.3 |
| 20:30 | 20:00 | 21:00 | 5.6 | 3.0 | 1.9 | 1.9 | 0.6 | 0.7 |
| 21:30 | 21:00 | 22:00 | 11.5 | 2.8 | 3.1 | 1.8 | 0.6 | 0.6 |
| 22:30 | 22:00 | 23:00 | 9.9 | 3.1 | 2.5 | 1.9 | 1.2 | 0.4 |
| 23:30 | 23:00 | 00:00 | 11.8 | 5.5 | 2.9 | 2.1 | 1.4 | 1.2 |
| 00:30 | 00:00 | 01:00 | 10.3 | 2.7 | 3.4 | 2.2 | 2.5 | 1.9 |
| 01:30 | 01:00 | 02:00 | 11.2 | 3.0 | 3.0 | 2.2 | 2.9 | 2.3 |
| 02:30 | 02:00 | 03:00 | 7.2 | 2.4 | 2.4 | 1.4 | 2.6 | 2.4 |
| 03:30 | 03:00 | 04:00 | 6.0 | 1.7 | 1.6 | 1.1 | 2.2 | 1.8 |
| 04:30 | 04:00 | 05:00 | 4.0 | 1.6 | 1.6 | 0.7 | 2.0 | 1.6 |
| 05:30 | 05:00 | 06:00 | 1.3 | 0.5 | 0.1 | 0.2 | 1.3 | 0.6 |

**Table S5. Biting rhythm of *An. albimanus* in Haiti (unpublished data communicated by Impoinvil).**

| Mean time | Start time | End time | Dame Marie Outside | Dame Marie Inside | Artibonite Outside | Artibonite Inside | Ouanam-inthe Outside | Ouanam-inthe Inside |
| --- | --- | --- | --- | --- | --- | --- | --- | --- |
| 17:30 | 17:00 | 18:00 |  |  | 0.1 | 0.8 | 0.2 | 0.3 |
| 18:30 | 18:00 | 19:00 | 0.25 | 0.22 | 0.1 | 0.3 | 0.0 | 0.0 |
| 19:30 | 19:00 | 20:00 | 0.35 | 0.21 | 0.9 | 1.0 | 0.2 | 0.1 |
| 20:30 | 20:00 | 21:00 | 0.37 | 0.22 | 1.2 | 1.0 | 0.2 | 0.1 |
| 21:30 | 21:00 | 22:00 | 0.33 | 0.10 | 1.3 | 0.8 | 0.1 | 0.1 |
| 22:30 | 22:00 | 23:00 | 0.36 | 0.13 | 1.1 | 1.3 | 0.0 | 0.0 |
| 23:30 | 23:00 | 00:00 | 0.32 | 0.17 | 1.9 | 1.6 | 0.0 | 0.1 |
| 00:30 | 00:00 | 01:00 | 0.13 | 0.08 | 1.0 | 1.1 | 0.0 | 0.0 |
| 01:30 | 01:00 | 02:00 | 0.14 | 0.12 | 1.0 | 0.7 | 0.0 | 0.0 |
| 02:30 | 02:00 | 03:00 | 0.09 | 0.03 | 0.9 | 0.7 | 0.0 | 0.0 |
| 03:30 | 03:00 | 04:00 | 0.15 | 0.12 | 0.8 | 0.6 | 0.1 | 0.0 |
| 04:30 | 04:00 | 05:00 | 0.36 | 0.17 | 1.4 | 0.6 | 0.0 | 0.0 |
| 05:30 | 05:00 | 06:00 | 0.23 | 0.18 | 0.5 | 0.5 | 0.0 | 0.0 |
| 06:30 | 06:00 | 07:00 | 0.25 | 0.25 |  |  |  |  |

**Table S6. Biting rhythm of *An. albimanus* in the Dominican Republic published by Mekuria (7).**

| Mean time | Start time | End time | Outside | Inside |
| --- | --- | --- | --- | --- |
| 18:30 | 18:00 | 19:00 | 38.0 | 5.1 |
| 19:30 | 19:00 | 20:00 | 87.2 | 20.0 |
| 20:30 | 20:00 | 21:00 | 126.3 | 42.8 |
| 21:30 | 21:00 | 22:00 | 138.4 | 25.9 |
| 22:30 | 22:00 | 23:00 | 128.2 | 7.8 |
| 23:30 | 23:00 | 00:00 | 107.9 | 5.9 |
| 00:30 | 00:00 | 01:00 | 102.9 | 0.7 |
| 01:30 | 01:00 | 02:00 | 83.0 | 1.6 |
| 02:30 | 02:00 | 03:00 | 67.1 | 1.4 |
| 03:30 | 03:00 | 04:00 | 61.3 | 2.5 |
| 04:30 | 04:00 | 05:00 | 75.8 | 1.4 |
| 05:30 | 05:00 | 06:00 | 24.3 | 1.4 |

**References**

1. Ricciardi ID. Definicion de los habitos alimentares de anofelinos de Guatemala y Republica Dominicana, por tecnicas de gel-precipitacion. Revista de Microbiologia. 1971;2:107-12.

2. Grieco JP, Achee NL, Andre RG, Roberts DR. Host feeding preferences of Anopheles species collected by manual aspiration, mechanical aspiration, and from a vehicle-mounted trap in the Toledo District, Belize, Central America. J Am Mosq Control Assoc. 2002;18(4):307-15.

3. Bangs MJ. The susceptibility and behavioral response of Anopheles albimanus Weidemann and Anopheles vestitipennis Dyar and Knab (Diptera: Culicidae) to insecticides in northern Belize, Central America. Ann Arbor: UMI; 1999.

4. Taylor RT. The ecology of *Anopheles albimanus* (Wied.) in Haiti. Mosquito News. 1966;26:393–7.

5. Hobbs JH, Sexton JD, St Jean Y, Jacques JR. The biting and resting behavior of Anopheles albimanus in northern Haiti. J Am Mosq Control Assoc. 1986;2(2):150-3.

6. Desenfant P. Rôle et bioécologie de *Anopheles albimanus* (Wiedemann, 1820) vecteur du paludisme en Haïti.: L’Université de Paris-Sud Centre d’Orsay; 1988.

7. Mekuria Y, Tidwell MA, Williams DC, Mandeville JD. Bionomic studies of the Anopheles mosquitoes of Dajabon, Dominican Republic. J Am Mosq Control Assoc. 1990;6(4):651-7.

8. Muirhead-Thomson RC, Mercier EC. Factors in malaria transmission by Anopheles albimanus in Jamaica. Part I. Ann Trop Med Parasitol. 1952;46(2):103-16.

9. Bown DN, Rodriguez MH, Arredondo-Jimenez JI, Loyola EG, Rodriguez MC. Intradomiciliary behavior of Anopheles albimanus on the coastal plain of southern Mexico: implications for malaria control. J Am Mosq Control Assoc. 1993;9(3):321-4.

10. Rodriguez MH, Bown DN, Arredondo-Jimenez JI, Villarreal C, Loyola EG, Frederickson CE. Gonotrophic cycle and survivorship of Anopheles albimanus (Diptera: Culicidae) in southern Mexico. J Med Entomol. 1992;29(3):395-9.

11. Rua GL, Quinones ML, Velez ID, Zuluaga JS, Rojas W, Poveda G, et al. Laboratory estimation of the effects of increasing temperatures on the duration of gonotrophic cycle of Anopheles albimanus (Diptera: Culicidae). Mem Inst Oswaldo Cruz. 2005;100(5):515-20.

12. Mekuria Y, Granados R, Tidwell MA, Williams DC, Wirtz RA, Roberts DR. Malaria transmission potential by Anopheles mosquitoes of Dajabon, Dominican Republic. J Am Mosq Control Assoc. 1991;7(3):456-61.

1. Estimated percent remaining in unsprayed house during the day, possibly affected by prior years of spraying [↑](#endnote-ref-1)
2. Indoor resting (caught immediately after flying to a resting place after biting indoors) of indoor biting in DDT-sprayed houses: 773/1332 in high density sprayed and138/273 in low density. In unsprayed houses, indoor resting was 100%. [↑](#endnote-ref-2)
3. Out of 88 mosquitoes, 15 did not land on indoor surfaces, 43 landed first flight on non-sprayable surfaces, 16 on walls and 14 on roofs. [↑](#endnote-ref-3)
4. Mean time required for a mosquito that has encountered a host to return to host-seeking, provided that the mosquito survives to search again [↑](#endnote-ref-4)
5. Interpolated for average temperature in Port-au-Prince of 28.1°C [↑](#endnote-ref-5)
6. Mean time from blood-meal to oviposition [↑](#endnote-ref-6)
