# Supplementary material for: Models of effectiveness of interventions against malaria transmitted by Anopheles albimanus
Source: Malar J. 2019 Aug 1;18:263. doi: 10.1186/s12936-019-2899-3 (PMC6670173; doi:10.1186/s12936-019-2899-3)
Supplement: Supplementary file 2 — Additional file 2. Parameterization of the mosquito gonotrophic cycle. [file 12936_2019_2899_MOESM2_ESM.pdf]

Parameter Values for Transmission Model

Nakul Chitnis, Thomas Smith, and Allan Schapira

6 September 2010

1 Introduction

In this short document, we define parameter values for the entomological transmission model defined in [1] with both, human and nonhuman, hosts. We define parameter values for *An. gambiae* s.s., *An. funestus*, and *An. arabiensis*; and determine effectiveness of insecticide-treated nets (ITNs), indoor residual spraying with DDT (IRS-DDT), indoor residual spraying with pyrethroids (lambda-cyhalothrin) (IRS-P), indoor residual spraying with bendiocarb (IRS-BC), and house-screening (HS). Table 1 describes the parameters used in the model and Table 2 describes the derived and field-measurable parameters in the model. Tables 3–8 define parameter values for the model in the absence of interventions for population sizes of 1000, 50 000, and 100 000, with and without nonhuman hosts. Tables 9–11 define the effects of interventions for the parameters that they affect for the three mosquito species.

We describe the derivations of the parameter values in the following sections. We assume no insecticide resistance in mosquitoes. We also assume that parameter values describing mosquito feeding cycle survival probabilities after the mosquitoes have contacted a host (human or nonhuman) do not vary between mosquito species. We model differences in species as differences in availabilities rates of human and nonhuman hosts and in the death rate while host-seeking. We note again that we define parameter values here for the autonomous model that does not include seasonality.

2 Baseline Parameter Values

The parameter values described in Tables 1 and 2 are for any number of nonhuman hosts, labeled i for $2 \leq i \leq n$ where there are $n-1$ nonhuman hosts. The subscript $i = 1$ is reserved for humans. The given parameter values in Tables 3–8 assume there is only one type of nonhuman hosts, so $n = 2$.

We do not determine a value for N_{v0} because a periodic sequence for N_{v0} is separately estimated for each seasonal EIR pattern. We use baseline values for all three mosquito species for θ_d, P_{B_1}, P_{C_1}, P_{D_1}, P_{E_1}, τ, and θ_s, from [2, Table 2] and the references therein. In the absence of better information, we assume

that the survival probabilities after contacting a host are the same for humans and nonhuman hosts, so for all three species,

$$P_{B_2} = P_{B_1}, \tag{1}$$
$$P_{C_2} = P_{C_1}, \tag{2}$$
$$P_{D_2} = P_{D_1}, \tag{3}$$
$$P_{E_2} = P_{E_1}. \tag{4}$$

2.1 Derivation of μ_{vA}, α_1, and α_i for $2 \le i \le n$

We derive parameter values for μ_{vA}, α_1, and α_i for $2 \le i \le n$ from data for A_0, χ, M, and ξ_i for $2 \le i \le n$. As we do not need to differentiate each individual nonhuman host, we do not derive separate values for α_i and N_i, but only consider their product, $N_i\alpha_i$ (or set $N_i = 1$). Note that while we derive the equations for an arbitrary number of nonhuman host types, in the specific examples in Tables 3–8, we assume there is only one type of nonhuman host so $n = 2$.

From [3, Table 1], we determine values for M and A_0 for the three mosquito species. From [4, Table 2] we determine values for χ for the three species, where we assume the existence of nonhuman hosts. For the scenarios where there are no nonhuman hosts, we assume $\chi = 1$. When there is only one type of nonhuman host, we set $\xi_2 = 1$ and do not show it in Tables 3–8.

We introduce a parameter, P_{A_h}, that is the probability of a mosquito encountering any nonhuman host on a given night,

$$P_{A_h} = \sum_{i=2}^{n} P_{A^i}. \tag{5}$$

We use ξ_i, $2 \le i \le n$, to denote the availability of nonhuman hosts, relative to other nonhuman hosts, that is,

$$\xi_i = \frac{P_{A^i}}{\sum_{k=2}^{n} P_{A^k}}, \tag{6}$$
$$= \frac{P_{A^i}}{P_{A_h}} \quad \text{for } 2 \le i \le n. \tag{7}$$

We note that,

$$\sum_{i=2}^{n} \xi_i = 1.$$

Solving for P_{A^i} in (7), gives us,

$$P_{A^i} = \xi_i P_{A_h} \quad \text{for } 2 \le i \le n. \tag{8}$$

While it may not necessarily be possible to find data to evaluate ξ_i, this is probably the best description we have at present to differentiate between the availability of different types of nonhuman hosts.

We know from [1, (8)] that,

$$P_f = M. \tag{9}$$

Similarly, we can show that the proportion of host-seeking (parous) mosquitoes that have waited at least one day since they laid eggs is P_A. Thus, the proportion of host-seeking (parous) mosquitoes that have laid eggs that same day is,

$$A_0 = 1 - P_A. \tag{10}$$

The definition of the human blood index as the proportion of resting mosquitoes that have fed on human blood (in that gonotrophic cycle) leads to,

$$\begin{aligned}\chi &= \frac{P_{A^1} P_{B_1} P_{C_1}}{P_{A^1} P_{B_1} P_{C_1} + \sum_{i=2}^{n} P_{A^i} P_{B_i} P_{C_i}}, \\ &= \frac{P_{A^1} P_{B_1} P_{C_1}}{P_{A^1} P_{B_1} P_{C_1} + P_{A_h} \sum_{i=2}^{n} \xi_i P_{B_i} P_{C_i}}. \end{aligned} \tag{11}$$

From [1, (2)], we have,

$$\begin{aligned}P_f &= \frac{P_{A^1} P_{B_1} P_{C_1} P_{D_1} P_{E_1} + \sum_{i=2}^{n} P_{A^i} P_{B_i} P_{C_i} P_{D_i} P_{E_i}}{1 - P_A} \\ &= \frac{P_{A^1} P_{B_1} P_{C_1} P_{D_1} P_{E_1} + P_{A_h} \sum_{i=2}^{n} \xi_i P_{B_i} P_{C_i} P_{D_i} P_{E_i}}{1 - P_A} \end{aligned} \tag{12}$$

Solving (10), (11), and (12) for P_A, P_{A^1}, and P_{A_h}, we get,

$$P_A = 1 - A_0, \tag{13}$$

$$P_{A^1} = \frac{A_0 P_f \chi \sum_{i=2}^{n} \xi_i P_{B_i} P_{C_i}}{P_{B_1} P_{C_1} \sum_{i=2}^{n} \left((\chi P_{D_1} P_{E_1} + (1-\chi) P_{D_i} P_{E_i}) \xi_i P_{B_i} P_{C_i} \right)}, \tag{14}$$

$$P_{A_h} = \frac{A_0 P_f (1-\chi)}{\sum_{i=2}^{n} \xi_i P_{B_i} P_{C_i} \left(\chi P_{D_1} P_{E_1} + (1-\chi) P_{D_i} P_{E_i} \right)}. \tag{15}$$

Then, from, (8),

$$P_{A^i} = \xi_i P_{A_h} \quad \text{for } 2 \le i \le n.$$

From the definitions of P_A and P_{A^i} for $1 \le i \le n$ in [1],

$$P_A = e^{-\left(\sum_{k=1}^{n} \alpha_k N_k + \mu_{vA}\right)\theta_d}, \tag{16}$$

$$P_{A^i} = \left(1 - e^{-\left(\sum_{k=1}^{n} \alpha_k N_k + \mu_{vA}\right)\theta_d}\right) \times \frac{\alpha_i N_i}{\sum_{k=1}^{n} \alpha_k N_k + \mu_{vA}}. \tag{17}$$

Using the change of variables,

$$x_i = \alpha_i N_i \quad \text{for } 1 \le i \le n, \tag{18}$$

$$y = \sum_{k=1}^{n} \alpha_k N_k + \mu_{vA}, \tag{19}$$

with inverse change of variables,

$$\alpha_i = \frac{x_i}{N_i} \quad \text{for } 1 \le i \le n, \tag{20}$$

$$\mu_{vA} = y - \left(\sum_{k=1}^{n} x_k\right), \tag{21}$$

we can rewrite (16)and (17) as,

$$P_A = e^{-y\theta_d}, \tag{22}$$

$$P_{A^i} = \left(1 - e^{-y\theta_d}\right)\frac{x_i}{y}. \tag{23}$$

Solving (22) and (23) for x_i, $1 \le i \le n$, and y, gives us,

$$x_i = \left(\frac{P_{A^i}}{1 - P_A}\right)\left(\frac{-\ln P_A}{\theta_d}\right) \quad \text{for } 1 \le i \le n, \tag{24}$$

$$y = \frac{-\ln P_A}{\theta_d}. \tag{25}$$

Substituting (24) and (25) into (20) and (21), we get,

$$\alpha_i = \frac{1}{N_i}\left(\frac{P_{A^i}}{1 - P_A}\right)\left(\frac{-\ln P_A}{\theta_d}\right) \quad \text{for } 1 \le i \le n, \tag{26}$$

$$\mu_{vA} = \left(\frac{1 - (P_A + \sum_{k=1}^{n} P_{A^k})}{1 - P_A}\right)\left(\frac{-\ln P_A}{\theta_d}\right), \tag{27}$$

for the three mosquito species.

3 Intervention-Modified Parameter Values

We show effects of interventions on entomological parameters for the three mosquito species in Tables 9–11. We assume that the effects of the interventions decay exponentially. The tables show the initial efficacy in proportional reduction and the half-life of the decay for the four parameters that can be affected by these interventions. When an intervention does not affect a given parameter, we set the reduction to 0 and do not give a value for the half-life.

We use half-life values for IRS-DDT, IRS-P, and IRS-BC from [5, Table 4], a survey of the use of insecticide across Africa. While there have been a few studies on the effective life time of insecticide-treated nets [6, 7], there is a wide range in the expected half-life of nets, depending on the type and treatment of nets. We use an accepted value of 3 years for the average half-life of nets. We assume this is the same for all three mosquito species. There are few studies on the half-life of the effectiveness of house-screening in reducing mosquito availability. From [8, Table 2], we see that for full screening, 58% of the screens are damaged after 6 months and 71% are damaged after 12 months. They describe the damage

to the screens but do not quantify the extent their effect on the reduction in mosquito entry rates. For simplicity, we assume that the house screening is repaired as necessary and that it's effect does not decay.

We use values for the proportional reduction for ITNs, IRS-DDT, and IRS-BC from [2, Table 3]. These numbers were calculated for *An. gambiae* s.l. so we use them for *An. gambiae* s.s. and *An. arabiensis.* In the absence of other information for *An. funestus* we assume the same effectiveness of the interventions.

We determine values for the effectiveness of IRS-P from data from [9]. For *An. gambiae* s.l. and *An. funestus*, they estimated,

D**:** The proportionate reduction in the number of mosquitoes entering treated huts,

F**:** The proportionate reduction in the number of mosquitoes feeding in treated huts, accounting for the reduction in mosquitoes that entered that hut,

K**:** The proportion of mosquitoes that died within one day of entering a treated hut, accounting for the reduction in mosquitoes that entered that hut,

for *An. gambiae* s.l.,

$$D = 0.2513, \tag{28a}$$
$$F = 0.07632, \tag{28b}$$
$$K = 0.7734, \tag{28c}$$

and for *An. funestus*,

$$D = 0.5857, \tag{29a}$$
$$F = 0.3712, \tag{29b}$$
$$K = 0.8252. \tag{29c}$$

Here we use the terminology, $i = 1$ to denote parameters for huts treated with the insecticide and $i = 2$ to denote parameters for untreated huts. Note that this is not the same as the rest of this document where we use $i = 1$ to denote humans and $i = 2$ to denote nonhuman hosts.

As their parameter K has already accounted for deterrence, making the assumption that the effects on mortality of lambda-cyhalothrin are the same for all mosquitoes as they are for resting mosquitoes, we see,

$$\frac{P_{D_1}}{P_{D_2}} = 1 - K, \tag{30}$$

so the proportionate reduction in P_{D_i} is K. Substituting parameter values for *An. gambiae* s.l. we get values for *An. gambiae* s.s. and *An. arabiensis.*

The ratio of the proportion of fed mosquitoes (who may or may not survive the resting period) in treated huts to the proportion of fed mosquitoes in untreated huts, is,

$$\varphi = \frac{N_v P_{A^1} P_{B_1} P_{C_1}}{N_v P_{A^2} P_{B_2} P_{C_2}}, \tag{31}$$

and in terms of the parameters in [9],

$$\varphi = (1 - D)(1 - F). \tag{32}$$

As the insecticide sprayed on the walls does not affect the biting survival probabilities of the mosquito,

$$P_{B_1} = P_{B_2},$$
$$P_{C_1} = P_{C_2}.$$

Therefore,

$$\varphi = \frac{P_{A^1}}{P_{A^2}}. \tag{33}$$

Assuming that the treated and untreated huts are available to the same population of mosquitoes, from [1], we have,

$$P_{A^1} = \frac{\alpha_1 N_1}{\sum_{k=1}^{N} \alpha_k N_k + \mu_{vA}}, \tag{34}$$

$$P_{A^2} = \frac{\alpha_2 N_2}{\sum_{k=1}^{N} \alpha_k N_k + \mu_{vA}}, \tag{35}$$

so,

$$\frac{P_{A^1}}{P_{A^2}} = \frac{\alpha_1 N_1}{\alpha_2 N_2}. \tag{36}$$

Since each hut has the same number of hosts in it,

$$N_1 = N_2, \tag{37}$$

so,

$$\frac{\alpha_1}{\alpha_2} = \frac{P_{A^1}}{P_{A^2}} = (1 - D)(1 - F). \tag{38}$$

Substituting (28) in (38), we can calculate the proportionate reduction in availability for *An. gambiae* s.s. and *An. arabiensis.* Similarly, substituting (29) in (38), we can calculate the proportionate reduction in availability for *An. funestus.*

[8, Table 2] shows that there was a 59% reduction in the number of mosquitoes (*An. gambiae* s.s., *An. melas*, and *An. arabiensis*) entering houses. We assume this is equivalent to a reduction in availability rate and applies to all three mosquito species considered here.

References

[1] Chitnis N, Smith T, Steketee R: **A mathematical model for the dynamics of malaria in mosquitoes feeding on a heterogeneous host population**. *Journal of Biological Dynamics* 2008, **2**(3):259–285.

[2] Chitnis N, Schapira A, Smith T, Steketee R: **Comparing the effectiveness of malaria vector-control interventions through a mathematical model**. *American Journal of Tropical Medicine and Hygiene* 2010, **83**(2):230–240.

[3] Charlwood JD, Smith T, Billingsley PF, Takken W, Lyimo EOK, Meuwissen JHET: **Survival and infection probabilities of anthropophagic anophelines from an area of high prevalence of *Plasmodium falciparum* in humans**. *Bulletin of Entomological Research* 1997, **87**:445–453.

[4] Kiszewski A, Mellinger A, Spielman A, Malaney P, Sachs SE, Sachs J: **A global index representing the stability of malaria transmission**. *American Journal of Tropical Medicine and Hygiene* 2004, **70**(5):486–498.

[5] Sadasivaiah S, Tozan Y, Breman JG: **Dichlorodiphenyltrichloroethane (DDT) for indoor residual spraying in Africa: How can it be used for malaria control?** *American Journal of Tropical Medicine and Hygiene* 2007, **77**(Suppl 6):249–263.

[6] Kilian A, Byamukama W, Pigeon O, Atieli F, Duchon S, Phan C: **Long-term field performance of a polyester-based long-lasting insecticidal mosquito net in rural Uganda**. *Malaria Journal* 2008, **7**(49).

[7] Lindblade KA, Dotson E, Hawley WA, Bayoh N, Williamson J, Mount D, Olang G, Vulule J, Slutsker L, Gimnig J: **Evaluation of long-lasting insecticidal nets after 2 years of household use**. *Tropical Medicine and International Health* 2005, **10**(11):1141–1150.

[8] Kirby MJ, Ameh D, Bottomley C, Green C, Jawara M, Milligan PJ, Snell PC, Conway DJ, Lindsay SW: **Effect of two different house screening interventions on exposure to malaria vectors and on anaemia in children in The Gambia: a randomised controlled trial**. *The Lancet* 2009, **374**(9694):998–1009.

[9] Tchicaya E, Others: **No Title Yet**. [In Preparation].

Table 1: Description of Parameters for Transmission Model. All instances of i in this table assume that $2 \leq i \leq n$.

n:	Number of different types of host types: $n \geq 2$.
N_{v0}:	Number of emerging mosquitoes that survive to the first feeding search per day.
N_1:	Total number of humans.
α_1:	Availability rate of each human to mosquitoes. This rate includes the reduction in availability of a host due to diversion.
$N_i\alpha_i$:	Total availability rate of all nonhuman hosts of type i.
μ_{vA}:	Per capita mosquito death rate while searching for a blood meal.
θ_d:	Maximum length of time that a mosquito searches for a host in one day if it is unsuccessful.
P_{B_1}:	Probability that a mosquito bites a human after encountering one.
P_{B_i}:	Probability that a mosquito bites a nonhuman host of type i after encountering one.
P_{C_1}:	Probability that a mosquito finds a resting place after biting a human.
P_{C_i}:	Probability that a mosquito finds a resting place after biting a nonhuman host of type i.
P_{D_1}:	Probability that a mosquito survives the resting phase after biting a human.
P_{D_i}:	Probability that a mosquito survives the resting phase after biting a nonhuman host of type i.
P_{E_1}:	Probability that a mosquito lays eggs and returns to host-seeking after biting a human.
P_{E_i}:	Probability that a mosquito lays eggs and returns to host-seeking after biting a nonhuman host of type i.
τ:	Time required for a mosquito that has encountered a host to return to host-seeking (provided that the mosquito survives to search again).
θ_s:	Duration of the extrinsic incubation period. This is the time required for sporozoites to develop in the mosquito.

Table 2: Description of Derived Parameters and Field-Measurable Quantities.
All instances of i in this table assume that $2 \leq i \leq n$.

P_A:	Probability that a mosquito does not find a host and does not die in one night of searching.
P_{A^1}:	Probability that a mosquito encounters a human on a given night.
P_{A_h}:	Probability that a mosquito encounters a nonhuman host on a given night.
P_{A^i}:	Probability that a mosquito encounters a nonhuman host of type i on a given night.
P_{df}:	Probability that a mosquito finds a host on a given night and then successfully completes the feeding cycle.
P_f:	Probability that a mosquito survives a feeding cycle.
P_{dif}:	Probability that a mosquito finds a host on a given night and then successfully completes the feeding cycle and gets infected.
P_{if}:	Probability that a mosquito survives a feeding cycle and gets infected.
M:	Parous rate. Proportion of host-seeking mosquitoes that have laid eggs at least once.
o_v:	Delayed oocyst rate. Proportion of host-seeking mosquitoes that are infected but not necessarily infective.
s_v:	Sporozoite rate. Proportion of host-seeking mosquitoes that are infective.
σ_1:	Human-biting rate. Number of mosquito bites that each human receives per unit of time.
Ξ_1:	Entomological inoculation rate: the number of infectious bites that one human receives per unit time.
Γ:	Vectorial capacity. The expected number of infectious bites on all hosts from mosquitoes infected by one "average" host in one unit of time.
γ:	Average expected lifespan of a mosquito.
χ:	Human blood index.
A_0:	Proportion of host-seeking parous mosquitoes that have laid eggs that day.
ξ_i:	Relative availability of nonhuman hosts of type i (to other nonhuman hosts).

Table 3: Parameter values for different *Anopheline* mosquito species in the absence of interventions with a human population of 1000 and the presence of animals.

	Units	*gambiae sl*	*gambiae ss*	*funestus*	*arabiensis*
N_{v0}:	Days^{-1}	-	-	-	-
N_1:	Animals	1000	1000	1000	1000
α_1:	Days^{-1}	0.0072	0.00085	0.0022	0.00079
$N_2\alpha_2$:	Animals $\times$ Days^{-1}	0	0.055	0.045	0.12
μ_{vA}:	Days^{-1}	1.6	0.24	0.65	0.24
θ_d:	Days	0.33	0.33	0.33	0.33
P_{B_1}:	1	0.95	0.95	0.95	0.95
P_{B_2}:	1	0.95	0.95	0.95	0.95
P_{C_1}:	1	0.95	0.95	0.95	0.95
P_{C_2}:	1	0.95	0.95	0.95	0.95
P_{D_1}:	1	0.99	0.99	0.99	0.99
P_{D_2}:	1	0.99	0.99	0.99	0.99
P_{E_1}:	1	0.88	0.88	0.88	0.88
P_{E_2}:	1	0.88	0.88	0.88	0.88
τ:	Days	3	3	3	3
θ_s:	Days	11	11	11	11
P_A:	1		0.687	0.384	0.687
P_{A^1}:	1		0.233	0.469	0.216
P_{A^2}:	1		0.0151	0.00957	0.320
P_{df}:	1		0.195	0.376	0.195
P_f:	1		0.623	0.611	0.623
P_{if}:	1				
M:	1		0.623	0.611	0.623
o_v:	1				
s_v:	1				
σ_1:	Days^{-1}				
Ξ_1:	Days^{-1}				
Γ:	Days^{-1}				
γ:	Days				
χ:	1		0.939	0.98	0.871
A_0:	1		0.313	0.616	0.313

Table 4: Parameter values for different *Anopheline* mosquito species in the absence of interventions with a human population of 1000 and the absence of animals.

	Units	*gambiae sl*	*gambiae ss*	*funestus*	*arabiensis*
N_{v0}:	Days^{-1}	-	-	-	-
N_1:	Animals	1000	1000	1000	1000
α_1:	Days^{-1}	0.0072	0.00090	0.0023	0.00090
$N_2\alpha_2$:	Animals $\times$ Days^{-1}	0	0	0	0
μ_{vA}:	Days^{-1}	1.6	0.24	0.65	0.24
θ_d:	Days	0.33	0.33	0.33	0.33
P_{B_1}:	1	0.95	0.95	0.95	0.95
P_{B_2}:	1	0.95	0.95	0.95	0.95
P_{C_1}:	1	0.95	0.95	0.95	0.95
P_{C_2}:	1	0.95	0.95	0.95	0.95
P_{D_1}:	1	0.99	0.99	0.99	0.99
P_{D_2}:	1	0.99	0.99	0.99	0.99
P_{E_1}:	1	0.88	0.88	0.88	0.88
P_{E_2}:	1	0.88	0.88	0.88	0.88
τ:	Days	3	3	3	3
θ_s:	Days	11	11	11	11
P_A:	1		0.687	0.384	0.687
P_{A^1}:	1		0.248	0.479	0.248
P_{A^2}:	1		0	0	0
P_{df}:	1		0.195	0.376	0.195
P_f:	1		0.623	0.611	0.623
P_{if}:	1				
M:	1		0.623	0.611	0.623
o_v:	1				
s_v:	1				
σ_1:	Days^{-1}				
Ξ_1:	Days^{-1}				
Γ:	Days^{-1}				
γ:	Days				
χ:	1		1	1	1
A_0:	1		0.313	0.616	0.313

Table 5: Parameter values for different *Anopheline* mosquito species in the absence of interventions with a human population of 50 000 and the presence of animals.

	Units	*gambiae sl*	*gambiae ss*	*funestus*	*arabiensis*
N_{v0}:	Days^{-1}	-	-	-	-
N_1:	Animals	1000	50 000	50 000	50 000
α_1:	Days^{-1}	0.0072	1.7×10^{-5}	4.4×10^{-5}	1.6×10^{-5}
$N_2\alpha_2$:	Animals × Days^{-1}	0	0.055	0.045	0.12
μ_{vA}:	Days^{-1}	1.6	0.24	0.65	0.24
θ_d:	Days	0.33	0.33	0.33	0.33
P_{B_1}:	1	0.95	0.95	0.95	0.95
P_{B_2}:	1	0.95	0.95	0.95	0.95
P_{C_1}:	1	0.95	0.95	0.95	0.95
P_{C_2}:	1	0.95	0.95	0.95	0.95
P_{D_1}:	1	0.99	0.99	0.99	0.99
P_{D_2}:	1	0.99	0.99	0.99	0.99
P_{E_1}:	1	0.88	0.88	0.88	0.88
P_{E_2}:	1	0.88	0.88	0.88	0.88
τ:	Days	3	3	3	3
θ_s:	Days	11	11	11	11
P_A:	1		0.687	0.384	0.687
P_{A^1}:	1		0.233	0.469	0.216
P_{A^2}:	1		0.0151	0.00957	0.320
P_{df}:	1		0.195	0.376	0.195
P_f:	1		0.623	0.611	0.623
P_{if}:	1				
M:	1		0.623	0.611	0.623
o_v:	1				
s_v:	1				
σ_1:	Days^{-1}				
Ξ_1:	Days^{-1}				
Γ:	Days^{-1}				
γ:	Days				
χ:	1		0.939	0.98	0.871
A_0:	1		0.313	0.616	0.313

Table 6: Parameter values for different *Anopheline* mosquito species in the absence of interventions with a human population of 50 000 and the absence of animals.

	Units	*gambiae sl*	*gambiae ss*	*funestus*	*arabiensis*
N_{v0}:	Days^{-1}	-	-	-	-
N_1:	Animals	1000	50 000	50 000	50 000
α_1:	Days^{-1}	0.0072	1.8×10^{-5}	4.5×10^{-5}	1.8×10^{-5}
$N_2\alpha_2$:	Animals $\times$ Days^{-1}	0	0	0	0
μ_{vA}:	Days^{-1}	1.6	0.24	0.65	0.24
θ_d:	Days	0.33	0.33	0.33	0.33
P_{B_1}:	1	0.95	0.95	0.95	0.95
P_{B_2}:	1	0.95	0.95	0.95	0.95
P_{C_1}:	1	0.95	0.95	0.95	0.95
P_{C_2}:	1	0.95	0.95	0.95	0.95
P_{D_1}:	1	0.99	0.99	0.99	0.99
P_{D_2}:	1	0.99	0.99	0.99	0.99
P_{E_1}:	1	0.88	0.88	0.88	0.88
P_{E_2}:	1	0.88	0.88	0.88	0.88
τ:	Days	3	3	3	3
θ_s:	Days	11	11	11	11
P_A:	1		0.687	0.384	0.687
P_{A^1}:	1		0.248	0.479	0.248
P_{A^2}:	1		0	0	0
P_{df}:	1		0.195	0.376	0.195
P_f:	1		0.623	0.611	0.623
P_{if}:	1				
M:	1		0.623	0.611	0.623
o_v:	1				
s_v:	1				
σ_1:	Days^{-1}				
Ξ_1:	Days^{-1}				
Γ:	Days^{-1}				
γ:	Days				
χ:	1		1	1	1
A_0:	1		0.313	0.616	0.313

Table 7: Parameter values for different *Anopheline* mosquito species in the absence of interventions with a human population of 100 000 and the presence of animals.

	Units	*gambiae sl*	*gambiae ss*	*funestus*	*arabiensis*
N_{v0}:	Days^{-1}	-	-	-	-
N_1:	Animals	1000	100 000	100 000	100 000
α_1:	Days^{-1}	0.0072	8.5×10^{-6}	2.2×10^{-5}	7.9×10^{-6}
$N_2\alpha_2$:	Animals $\times$ Days^{-1}	0	0.055	0.045	0.12
μ_{vA}:	Days^{-1}	1.6	0.24	0.65	0.24
θ_d:	Days	0.33	0.33	0.33	0.33
P_{B_1}:	1	0.95	0.95	0.95	0.95
P_{B_2}:	1	0.95	0.95	0.95	0.95
P_{C_1}:	1	0.95	0.95	0.95	0.95
P_{C_2}:	1	0.95	0.95	0.95	0.95
P_{D_1}:	1	0.99	0.99	0.99	0.99
P_{D_2}:	1	0.99	0.99	0.99	0.99
P_{E_1}:	1	0.88	0.88	0.88	0.88
P_{E_2}:	1	0.88	0.88	0.88	0.88
τ:	Days	3	3	3	3
θ_s:	Days	11	11	11	11
P_A:	1		0.687	0.384	0.687
P_{A^1}:	1		0.233	0.469	0.216
P_{A^2}:	1		0.0151	0.00957	0.320
P_{df}:	1		0.195	0.376	0.195
P_f:	1		0.623	0.611	0.623
P_{if}:	1				
M:	1		0.623	0.611	0.623
o_v:	1				
s_v:	1				
σ_1:	Days^{-1}				
Ξ_1:	Days^{-1}				
Γ:	Days^{-1}				
γ:	Days				
χ:	1		0.939	0.98	0.871
A_0:	1		0.313	0.616	0.313

Table 8: Parameter values for different *Anopheline* mosquito species in the absence of interventions with a human population of 100 000 and the absence of animals.

	Units	*gambiae sl*	*gambiae ss*	*funestus*	*arabiensis*
N_{v0}:	Days^{-1}	-	-	-	-
N_1:	Animals	1000	100 000	100 000	100 000
α_1:	Days^{-1}	0.0072	9.0×10^{-6}	$2.3 \times 10{-}5$	9.0×10^{-6}
$N_2\alpha_2$:	Animals $\times$ Days^{-1}	0	0	0	0
μ_{vA}:	Days^{-1}	1.6	0.24	0.65	0.24
θ_d:	Days	0.33	0.33	0.33	0.33
P_{B_1}:	1	0.95	0.95	0.95	0.95
P_{B_2}:	1	0.95	0.95	0.95	0.95
P_{C_1}:	1	0.95	0.95	0.95	0.95
P_{C_2}:	1	0.95	0.95	0.95	0.95
P_{D_1}:	1	0.99	0.99	0.99	0.99
P_{D_2}:	1	0.99	0.99	0.99	0.99
P_{E_1}:	1	0.88	0.88	0.88	0.88
P_{E_2}:	1	0.88	0.88	0.88	0.88
τ:	Days	3	3	3	3
θ_s:	Days	11	11	11	11
P_A:	1		0.687	0.384	0.687
P_{A^1}:	1		0.248	0.479	0.248
P_{A^2}:	1		0	0	0
P_{df}:	1		0.195	0.376	0.195
P_f:	1		0.623	0.611	0.623
P_{if}:	1				
M:	1		0.623	0.611	0.623
o_v:	1				
s_v:	1				
σ_1:	Days^{-1}				
Ξ_1:	Days^{-1}				
Γ:	Days^{-1}				
γ:	Days				
χ:	1		1	1	1
A_0:	1		0.313	0.616	0.313

Table 9: Parameter values for the effects of different interventions on mosquito gonotrophic cycle parameters. This table shows the initial efficacy in proportionate reduction in the entomological parameter, and its half-life, that the given intervention causes for *Anopheles gambiae* s.s. with insecticide-treated nets (ITNs), indoor residual spraying with DDT (IRS-DDT), indoor residual spraying with lambda-cyhalothrin (IRS-P), indoor residual spraying with bendiocarb (IRS-BC), and house-screening (HS). The proportional reduction is dimensionless while the half-life is measured in years.

		ITNs	IRS-DDT	IRS-P	IRS-BC	HS
α_1	Reduction:	0.44	0.56	0.31	0	0.59
α_1	Half-life:	3	0.5	0.25	-	∞
P_{B_1}	Reduction:	0.27	0	0	0	0
P_{B_1}	Half-life:	3	-	-	-	-
P_{C_1}	Reduction:	0.27	0	0	0	0
P_{C_1}	Half-life:	3	-	-	-	-
P_{D_1}	Reduction:	0	0.24	0.77	0.81	0
P_{D_1}	Half-life:	-	0.5	0.25	0.17	-

Table 10: Parameter values for the effects of different interventions on mosquito gonotrophic cycle parameters. This table shows the initial efficacy in proportionate reduction in the entomological parameter, and its half-life, that the given intervention causes for *Anopheles arabiensis* with insecticide-treated nets (ITNs), indoor residual spraying with DDT (IRS-DDT), indoor residual spraying with lambda-cyhalothrin (IRS-P), indoor residual spraying with bendiocarb (IRS-BC), and house-screening (HS). The proportional reduction is dimensionless while the half-life is measured in years.

		ITNs	IRS-DDT	IRS-P	IRS-BC	HS
α_1	Reduction:	0.44	0.56	0.31	0	0.59
α_1	Half-life:	3	0.5	0.25	-	∞
P_{B_1}	Reduction:	0.27	0	0	0	0
P_{B_1}	Half-life:	3	-	-	-	-
P_{C_1}	Reduction:	0.27	0	0	0	0
P_{C_1}	Half-life:	3	-	-	-	-
P_{D_1}	Reduction:	0	0.24	0.77	0.81	0
P_{D_1}	Half-life:	-	0.5	0.25	0.17	-

Table 11: Parameter values for the effects of different interventions on mosquito gonotrophic cycle parameters. This table shows the initial efficacy in proportionate reduction in the entomological parameter, and its half-life, that the given intervention causes for *Anopheles funestus* with insecticide-treated nets (ITNs), indoor residual spraying with DDT (IRS-DDT), indoor residual spraying with lambda-cyhalothrin (IRS-P), indoor residual spraying with bendiocarb (IRS-BC), and house-screening (HS). The proportional reduction is dimensionless while the half-life is measured in years.

		ITNs	IRS-DDT	IRS-P	IRS-BC	HS
α_1	Reduction:	0.44	0.56	0.74	0	0.59
α_1	Half-life:	3	0.5	0.25	-	∞
P_{B_1}	Reduction:	0.27	0	0	0	0
P_{B_1}	Half-life:	3	-	-	-	-
P_{C_1}	Reduction:	0.27	0	0	0	0
P_{C_1}	Half-life:	3	-	-	-	-
P_{D_1}	Reduction:	0	0.24	0.83	0.81	0
P_{D_1}	Half-life:	-	0.5	0.25	0.17	-
