# Supplementary material for: Models of effectiveness of interventions against malaria transmitted by Anopheles albimanus
Source: Malar J. 2019 Aug 1;18:263. doi: 10.1186/s12936-019-2899-3 (PMC6670173; doi:10.1186/s12936-019-2899-3)
Supplement: Supplementary file 3 — Additional file 3. Parameterization of intervention effects. [file 12936_2019_2899_MOESM3_ESM.docx]

Parameterization of intervention effects

Table of Contents

[1 Vectorial capacity 3](#_Toc499736089)

[2 Effects of ITNs 4](#_Toc499736090)

[3 Effects of IRS 14](#_Toc499736091)

[4 References 18](#_Toc499736092)

Table of Figures

[**Figure S1. The proportion of mosquitoes being deterred from entering huts depending on the insecticide concentration in the net in the hut.** 7](#_Toc499736093)

[**Figure S2. The proportion of mosquitoes attacking (resulting in feeding and / or dying) inside huts with nets depending on the insecticide concentration and holed surface area in the net** 8](#_Toc499736094)

[**Figure S3. The proportion of mosquitoes being killed before feeding depending on the insecticide concentration and holed surface area in the net** 9](#_Toc499736095)

[**Figure S4. The proportion of mosquitoes being killed after feeding depending on the insecticide concentration and holed surface area in net** 10](#_Toc499736096)

[**Figure S5. The overall insecticidal effect on mosquitoes of a net depending on the insecticide concentration and holed surface area** 11](#_Toc499736097)

[**Figure S6. The personal protection provided by a net against mosquito bites depending on the insecticide concentration and holed surface area** 12](#_Toc499736098)

[**Figure S7. The overall protection against feeding provided by a net depending on the insecticide concentration and holed surface area** 13](#_Toc499736099)

[**Figure S8. The proportional impact on vectorial capacity of mosquitoes depending on the insecticide concentration and holed surface area in the nets in a population with a use coverage of 80% of identical nets, assuming all biting occurs when people are asleep** 14](#_Toc499736100)

[**Figure S9. Effect of indoor residual spray on mosquitoes** 18](#_Toc499736101)

Table of Tables

[**Table S1: Entomological parameter values** 3](#_Toc499736102)

[**Table S2: Experimental hut results with *An. albimanus* in in coastal Chiapas, Mexico published by Arredondo-Jimenez and colleagues (CITE YEAR 1997)** 4](#_Toc499736103)

[**Table S3: Experimental hut results with *An. gambiae* in Malanville published by N’Guessan and colleagues (CITE)** 5](#_Toc499736104)

[**Table S4: LLIN effect parameter values** 6](#_Toc499736105)

[**Table S5: Experimental hut results with *An. gambiae* in Natsini grouped in four catagories, interpreted from data published by Kuhlow (1962 CITE YEAR)** 15](#_Toc499736106)

# Vectorial capacity

## Simulation of vectorial capacity

Simulations of vectorial capacity (VC) were performed using an entomological model (1) which was implemented in the R software platform. In each simulation, the total population consisted of 2,000 individuals. The number of humans out of these was 1,000 for the main simulations, but after calculating the mortality while host seeking in the absence of interventions ($\mu_{\upsilon A}$) the number of humans was varied between 5 and 2000 to investigate the influence of the presence of animals.

The entomological model parameters used for *Anopheles gambiae* (see Table 1) were identical to those described by Chitnis and colleagues (2) in Table 3 for *Anopheles gambiae* s.s. The mosquito emergence (Nv0) was fixed at 25000 and $\mu_{\upsilon A}$ and $\alpha$-values were calculated as described by Chitnis and colleagues (2) for a scenario without intervention.

**Table S1: Entomological parameter values**

| Parameter | *Anopheles albimanus* | *Anopheles gambiae* s.s. |
| --- | --- | --- |
| Nh | 1000 | 1000 |
| Nc | 1 | 1 |
| M | 0.484 | 0.623 |
| Chi | 0.054 | 0.939 |
| A_0 | 0.405 | 0.313 |
| tau | 3 | 3 |
| theta_s | 11 | 11 |
| PB.h.u, PB.c | 0.95 | 0.95 |
| PC.h.u, PC.c | 0.95 | 0.95 |
| PD.h.u, PD.c | 0.99 | 0.99 |
| PE.h.u, PE.c | 0.88 | 0.88 |
| theta_d | 0.33 | 0.33 |
| N_v0 | 25000 | 25000 |
|  |  |  |

The addition ‘h.u’ indicates an unprotected human, c indicates an (unprotected) animal. All other parameter names are as described by Chitnis and colleagues in (2).

## Effects of interventions

For the simulation of the impact of interventions against adult mosquitoes, the entomological model requires parameterization of the effects on the parameters $\alpha$ (availability of the host to mosquito attack), PB (the proportion initially surviving attack and progressing to blood feeding) and PC (the proportion surviving after blood feeding and entering the resting stage). Instead of in terms of effects on PC, effects could alternatively be parameterized in terms of change in PD or PE. These effects can be parameterized in terms of reduction of these parameters, as aversion of mosquitoes, proportion killed before feeding (post-prandial killing), and proportion killed after feeding (post-prandial killing).

The period between deployments (a half year for indoor residual spray (IRS) and three years for insecticide treated nets (ITNs)) was divided into 100 equal intervals, and at each interval mid-point, the effect at that time point was calculated from the assumed relationship between intervention effect and time. The VC was then calculated at that time point, depending on the coverage of the intervention. For ITNs, due to attrition, this coverage was reduced over the three year period.

## Proportion of vectorial capacity averted

The VC in scenarios with interventions was compared to the VC in a scenario without any LLINs, and the impact on VC was calculated as the proportion of VC in the absence of the intervention that was averted by the intervention. This was done for each time point over the duration of the period between deployments, and then averaged over all time points.

# Effects of ITNs

Effects of ITNs depending on time since distribution were modelled by first parameterizing effects of ITNs on mosquitoes depending on the total holed surface area in ITNs and on the insecticide content remaining in the ITNs. Subsequently, the insecticide content was assumed to follow an exponential decay curve with a half-life of 1.5 years (3). The curve of the total holed surface area with time was fitted as a parabolic growth curve to the mean holed area over time since distribution in PermaNet 2.0 nets (including the intact ones) estimated to be 69.9 cm^2^, 162.6 cm^2^ and 558 cm^2^ for years 1, 2 and 3, respectively (4). The ITNs coverage was modelled to attritite following a ‘smooth-compact’ decay curve with a half-life of four years (3).

## Data

**Lambdacyhalothrin ITN effects on *An. albimanus***

Arredondo-Jimenez and colleagues (5) studied intact ITNs with lambdacyhalothrin 30 mg/m^2^ 0–17 weeks post impregnation. Selected results are summarized in Table 2.

**Table S2: Experimental hut results with *An. albimanus* in in coastal Chiapas, Mexico published by Arredondo-Jimenez and colleagues (5)**

| Treatment | Total | Fed (%) | Dead (%) |
| --- | --- | --- | --- |
| Untreated net (two sleeping outside) | 595 | 24.0 | 6.0 |
| Untreated net (two sleeping inside) | 592 | 23.6 | 8.0 |
| Nylon ITN 30 mg/m^2^ lambdacyhalothrin, two sleeping outside | 592 | 14.9 | 16.0 |
| Nylon ITN 30 mg/m^2^ lambdacyhalothrin, two sleeping inside | 524 | 7.9 | 16.0 |

Comparing the untreated net with the ITN, the deterrence from approaching was 11.9% for two inside net, and 0.5% for two outside net.

It is unknown how many of the fed were dead. For simplicity, we can presume independence, which was observed in a study by Randriamaherijaona and colleagues (6). Note again the higher proportion attacking in ITN than in control. ITNs protect more against biting than IRS (as expected), but have slightly less killing than IRS. ITN effects against *An. albimanus* were simulated assuming a target concentration of 30 mg/m^2^.

**Lambdacyhalothrin ITN effects on** ***An. gambiae***

N’Guessan and colleagues (7) tested an untreated net and a net impregnated with lambdacyhalothrin (18 mg/m^2^), as well as lambdacyhalothrin IRS on walls of a hut (30 mg/m^2^) and a control hut sprayed with water, against *An. gambiae* (95% *An. gambiae* s.s.) in Malanville (11.8633° N, 3.3842° E), northern Benin. Both nets had each 80 holes, each measuring 2 × 2 cm. Selected results are summarized in Table 3.

**Table S3: Experimental hut results with *An. gambiae* in Malanville published by N’Guessan and colleagues** (7)

| Treatment | Total | Fed (%) | Dead (%) |
| --- | --- | --- | --- |
| Untreated net | 363 | 77.7 | 3.6 |
| ITN 18 mg/m^2^ lambdacyhalothrin | 267 | 3.0 | 98.5 |
| Walls sprayed with water | 498 | 93.8 | 1.4 |
| Walls sprayed with 30 mg/m^2^ lambdacyhalothrin | 395 | 69.6 | 72.1 |

For this parameterization, it was assumed that IRS with 30 mg/m^2^ was equivalent in effect of a completely torn (holed surface area 192000 cm^2^) ITN with 30 mg/m^2^ lambdacyhalothrin, and that the probability of dying was equal for fed and unfed mosquitoes. ITN effects against *An. gambiae* were simulated assuming a target concentration of 18 mg/m^2^.

## Effects of ITNs depending on the holed surface area and insecticide content

For the simulation of the impact of ITN-use, the availability of hosts protected by an ITN for attack by mosquitos be split into two components: the proportion entering houses (as a fraction is possibly deterred from entering by chemicals emanating from the houses), which is purely a function of insecticide concentration, and the proportion attacking out of those that entered houses (a function of chemical concentration and holed surface area of the LLIN).

The effects of logarithmically transformed insecticide concentration and logarithmically transformed holed surface area on the outcomes ‘proportion attacking’ (PAtt), ‘proportion being killed before feeding’ (PBmu), and ‘proportion or being killed after feeding’ (PCmu) were estimated in Bayesian binomial regression models with logit link function, and included an interaction term for holed surface area and insecticide concentration. For the ‘proportion entering huts’ (PEnt), only logarithmically transformed insecticide concentration was an explanatory variable. For PEnt, the intercept was fixed at logit(0.99)=4.59512, assuming that 99% of mosquitoes enter houses without any emanating insecticide (instead of an assumption of 100% entering without insecticide, which would give problems with logit transformation). The intercept for PEnt was fixed, because there was only one data point available. The insecticide concentration was logarithmically transformed because it was assumed that the effect saturates with increasing insecticide content.

Also for the estimation of the post-prandial killing effect of ITNs with *An. gambiae*, the intercept was fixed at logit(0.01). Results of the logistic regression models are shown in Table 4, and the relationships are plotted in Figure 1 to Figure 4. Figure 5 illustrates the overall insecticidal effect (8), Figure 6 illustrates the personal protection, and Figure 8 shows the proportional impact on VC in hypothetical situations where 80%of the population use identical LLINs. At 80% coverage, the plot of impact on VC mostly resembles that of the plot of personal protection. It is noteworthy that the plots of overall insecticidal effect (Figure 5) on *An. gambiae* s.l. shows a peak at an insecticide concentration of about 150 mg/m^2^, after which the effect declines. This is because the deterrence from hut entry (Figure 1) increasingly prevents mosquitoes from being killed with increasing concentration.

Note that the proportion of *An. albimanus* mosquitoes attacking with a person under a lambda-cyhalothrin ITN (Figure 2) is larger than with an unprotected person (maximum holed surface area and without insecticide), and decreases as the net gains holes and loses insecticide. This occurs because in the study with *An. albimanus*, curiously, the proportion of mosquitoes (out of those entering a hut) that were attacking people inside untreated nets (with these mosquitoes ending up dead and / or fed) was similar to the proportion attacking people outside an untreated net (or outside a treated net), where many mosquitoes simply entered the hut, did not feed, and exited unfed (and alive).

The overall insecticidal effect (8) (Figure 5) is the probability that a mosquito that approaches a hut with a treatment ends up dead, due to the treatment alone, and not due to background mortality. Mosquitoes that are deterred from entry or repelled from attacking the person in the treated hut are subject to the ‘background’ mortality associated with approaching a person in a hut without a treatment. Similarly, the overall protection against feeding (Figure 7) is the probability that a mosquito that encounters a person protected by a net is inhibited from feeding (assuming that a deterred / repelled mosquito may feed on another unprotected host), relative to a mosquito that encounters an unprotected host.

**Table S4: LLIN effect parameter values**

| Parameter | Lambdacyhalothrin ITN on *An. albimanus* | Lambdacyhalothrin ITN on *An. gambiae* s.s Malanville |
| --- | --- | --- |
| beta0.PEnt | logit(0.99) | logit(0.99) |
| beta1.PEnt | -0.754 | -1.212 |
| beta0.PAtt | -0.861 | -0.020 |
| beta1.PAtt | -0.005 | 0.229 |
| beta2.PAtt | 0.253 | 2.006 |
| beta3.PAtt | -0.021 | -0.173 |
| beta0.PBmu | -1.370 | -2.914 |
| beta1.PBmu | -0.025 | -0.313 |
| beta2.PBmu | 0.893 | 3.864 |
| beta3.PBmu | -0.035 | -0.184 |
| beta0.PCmu | -2.501 | logit(0.01) (fixed) |
| beta1.PCmu | -0.021 | 0.046 |
| beta2.PCmu | 0.683 | 1.470 |
| beta3.PCmu | -0.031 | 0 (fixed) |
| HolesMax | 192000 | 192000 |

The regression model used is logit(PXxx) = beta0 + beta1 * log(Holes + 1) + scaling-factor * beta2 * log(Chem + 1) + beta3 * scaling-factor * log(Holes + 1) * log(Chem + 1), with the PXxx the proportion of mosquitoes entering (PEnt), attacking (PAtt), being killed before feeding (PBmu), or being killed after feeding (PCmu). Holes is the holed surface area in cm^2^, Chem is the insecticide concentration in mg/m^2^. HolesMax is the assumed total surface of an LLIN, and is used to calculate the effects for an unprotected human.

**
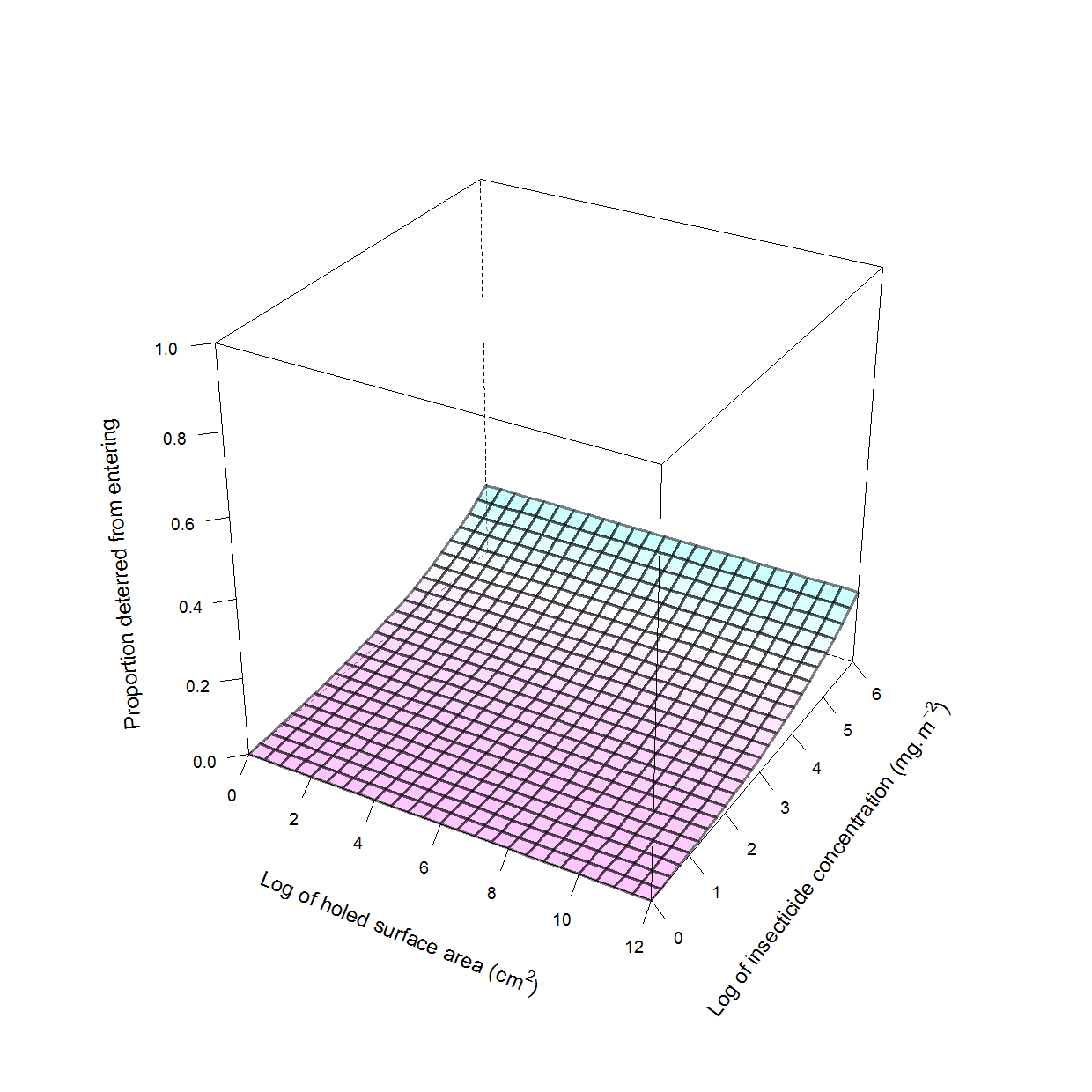
**
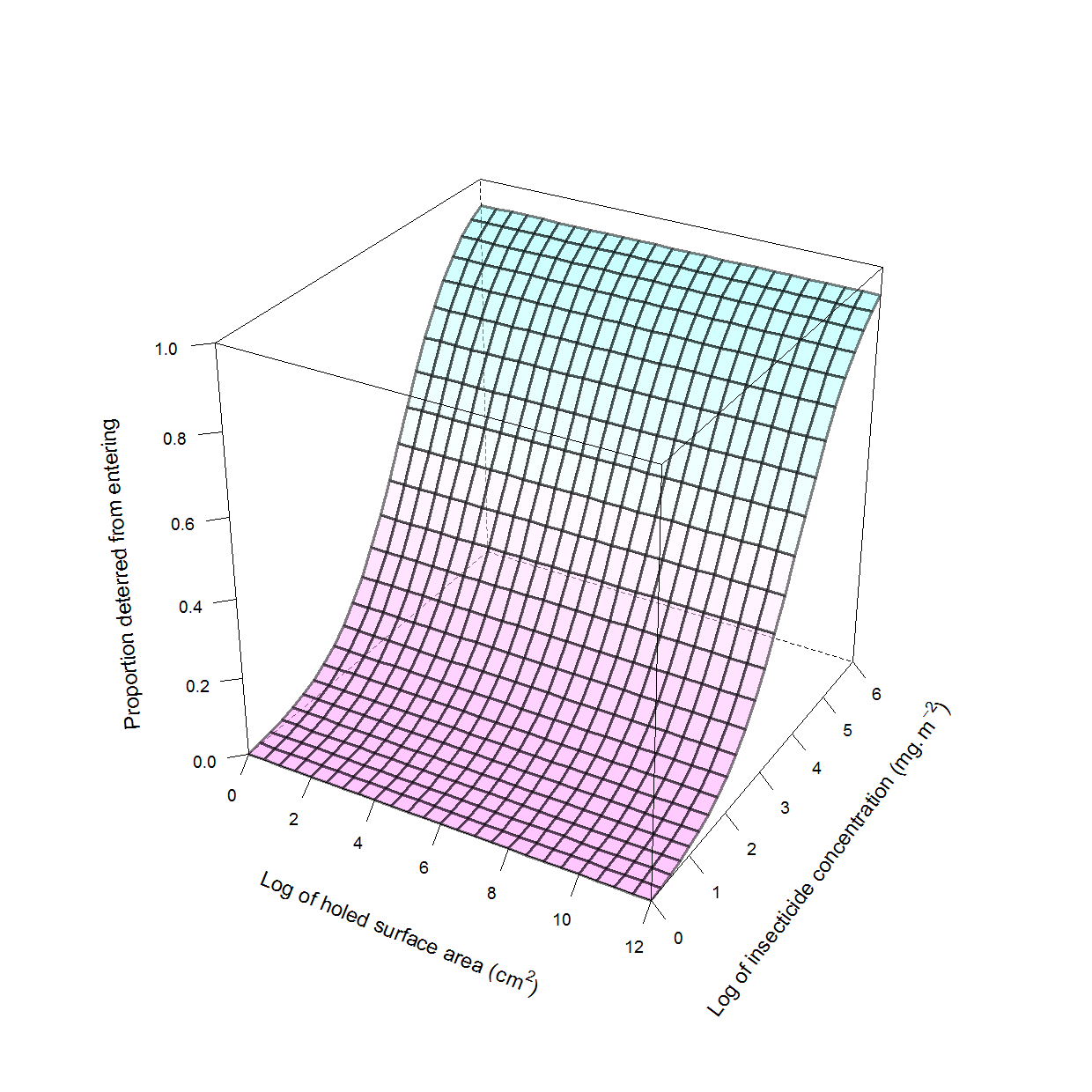


**Figure S1. The proportion of mosquitoes being deterred from entering huts depending on the insecticide concentration in the net in the hut.**

Left panel: *An. albimanus* with lambdacyhalothrin ITNs; right panel: *An. gambiae* s.l. with lambdacyhalothrin ITNs. The deterrence is independent of the holed surface area in the LLIN, but this is shown for consistency with subsequent figures.


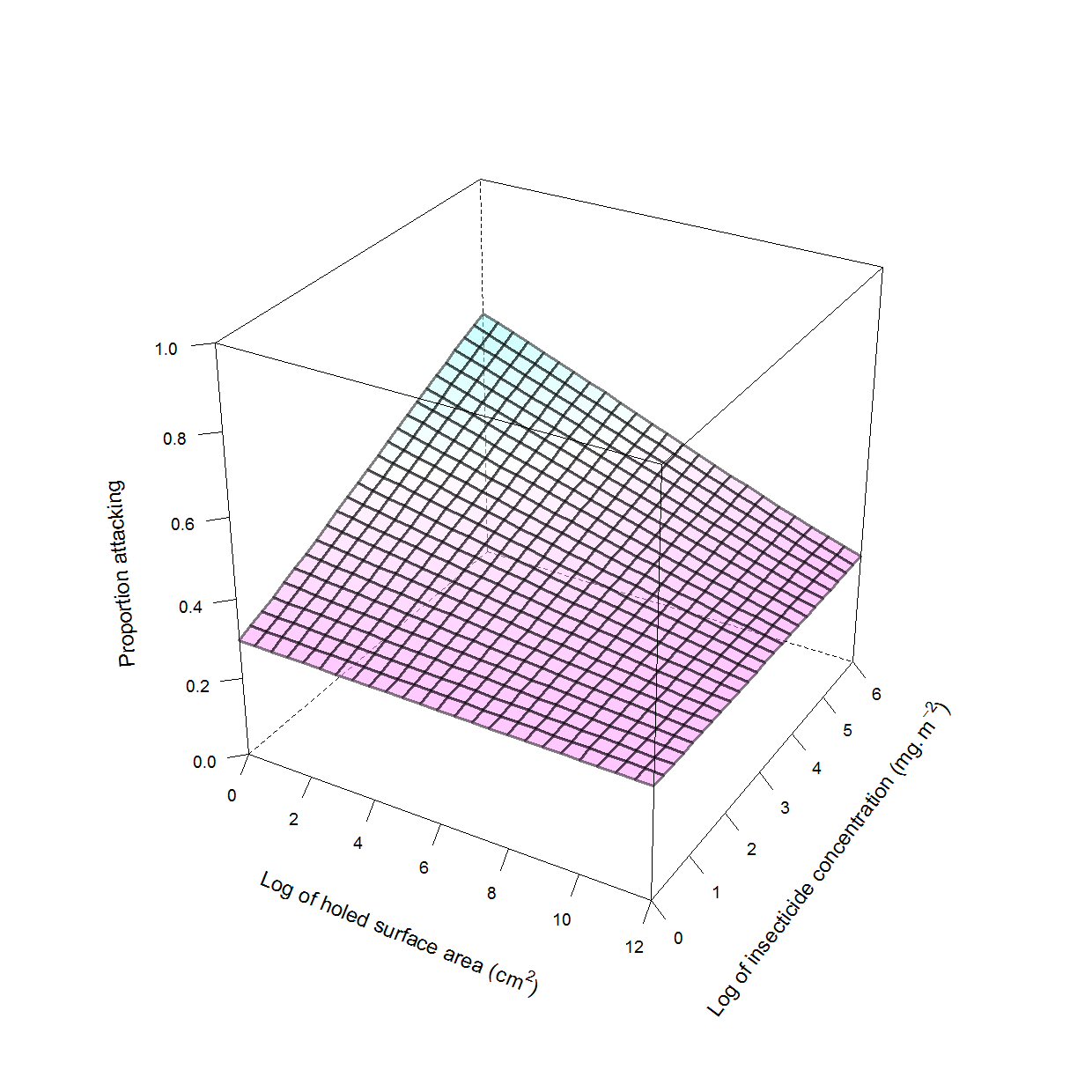

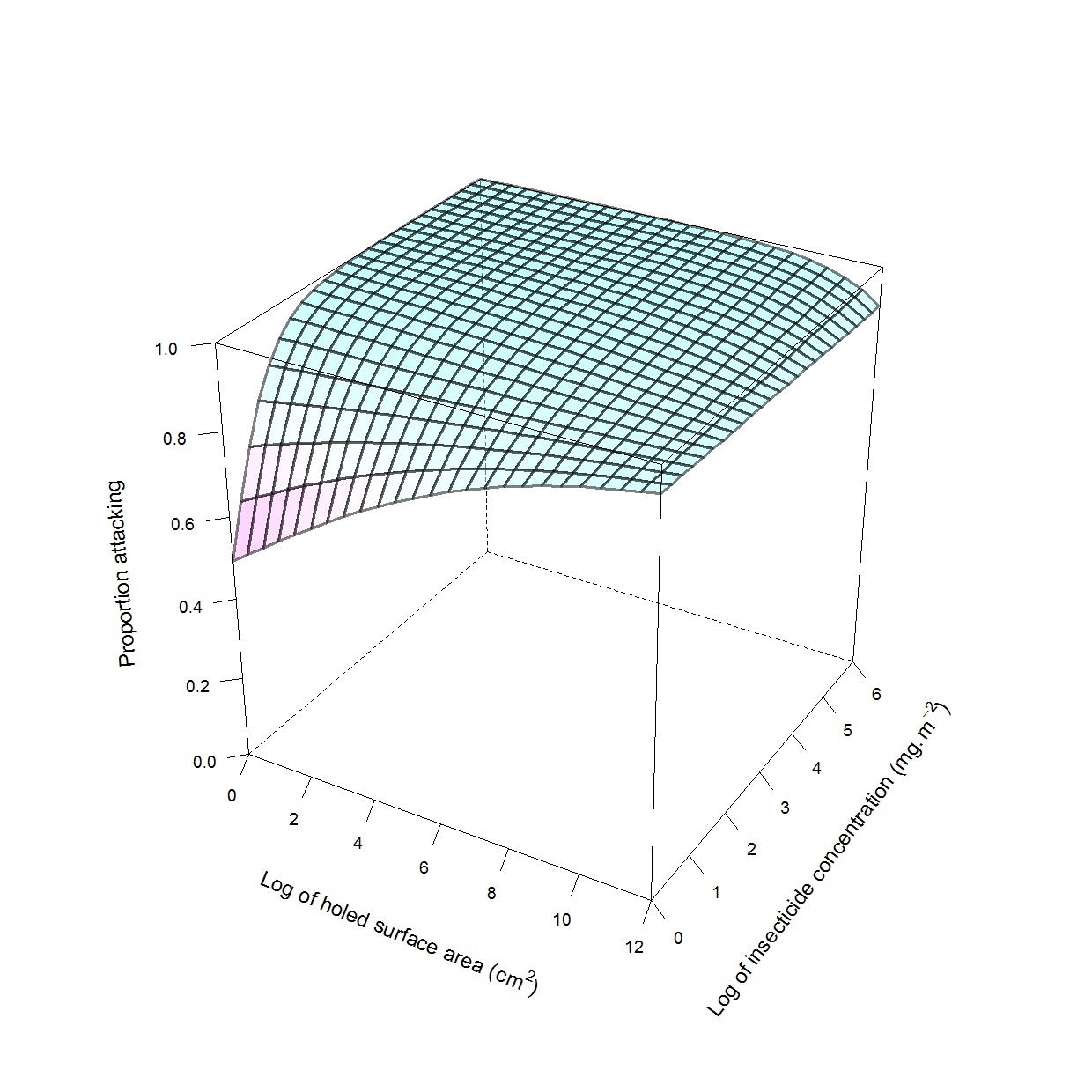


**Figure S2. The proportion of mosquitoes attacking (resulting in feeding and / or dying) inside huts with nets depending on the insecticide concentration and holed surface area in the net**

Left panel: *An. albimanus* with lambdacyhalothrin ITNs; right panel: *An. gambiae* s.l. with lambdacyhalothrin ITNs.

**
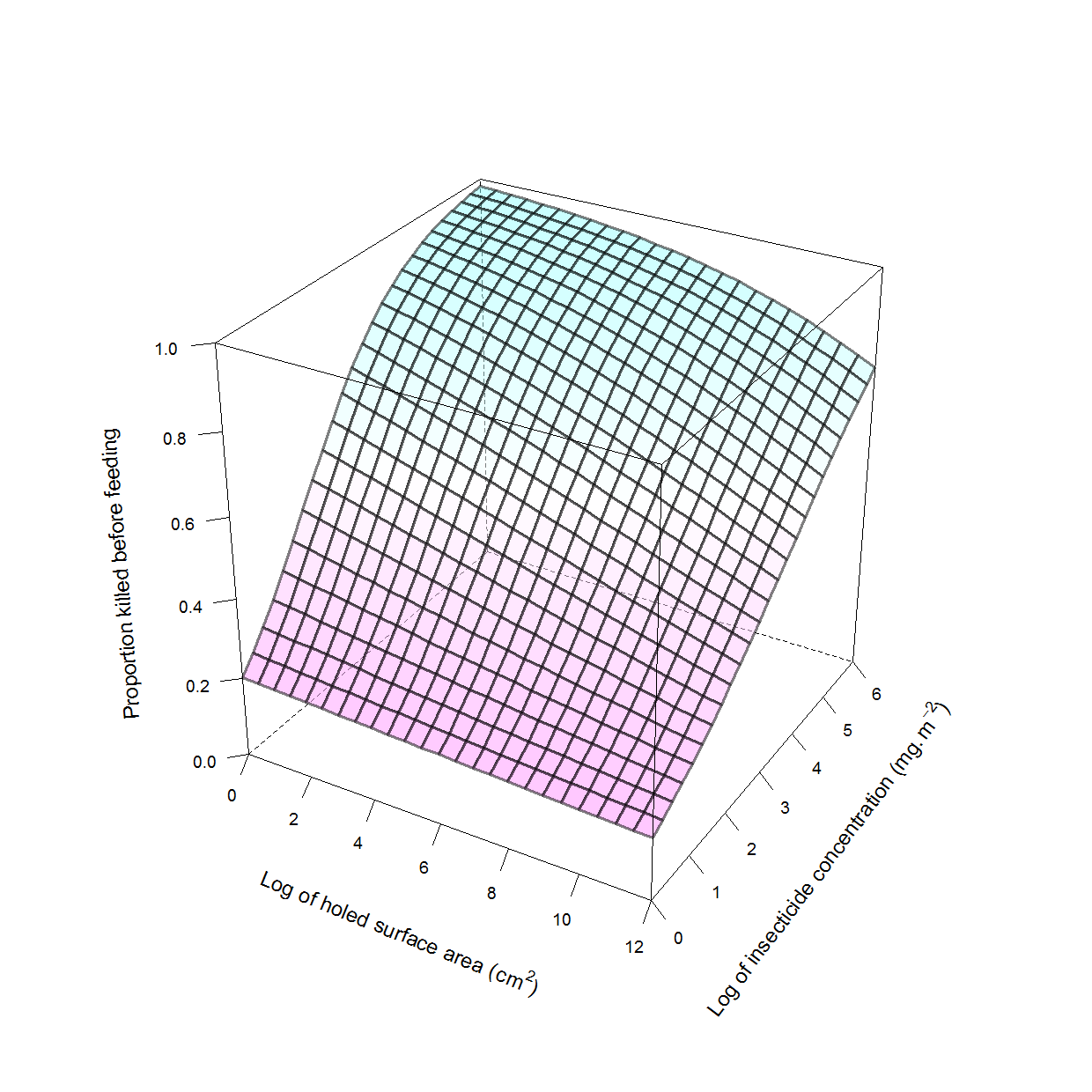

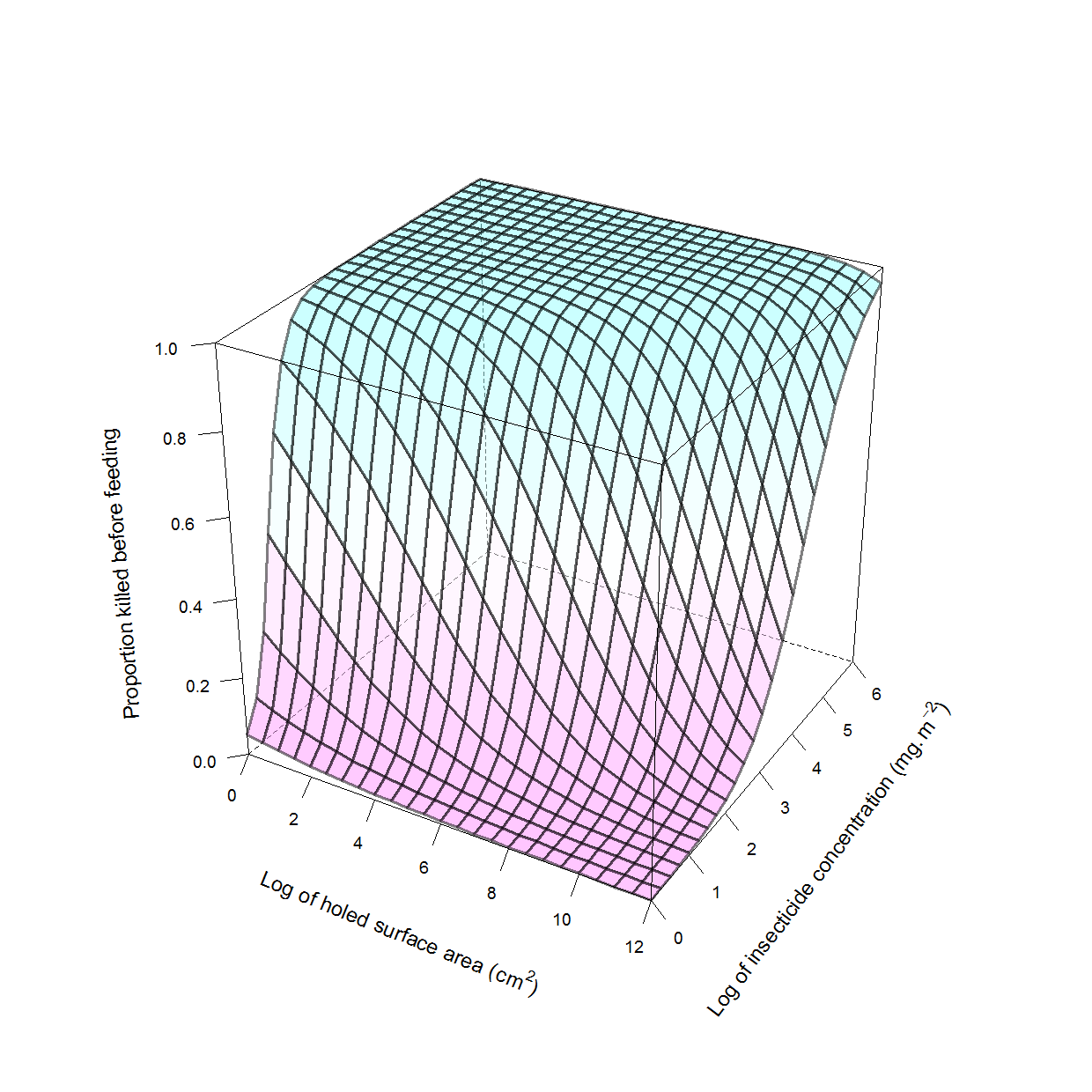
**

**Figure S3. The proportion of mosquitoes being killed before feeding depending on the insecticide concentration and holed surface area in the net**

Left panel: *An. albimanus* with lambdacyhalothrin ITNs; right panel: *An. gambiae* s.l. with lambdacyhalothrin ITNs.


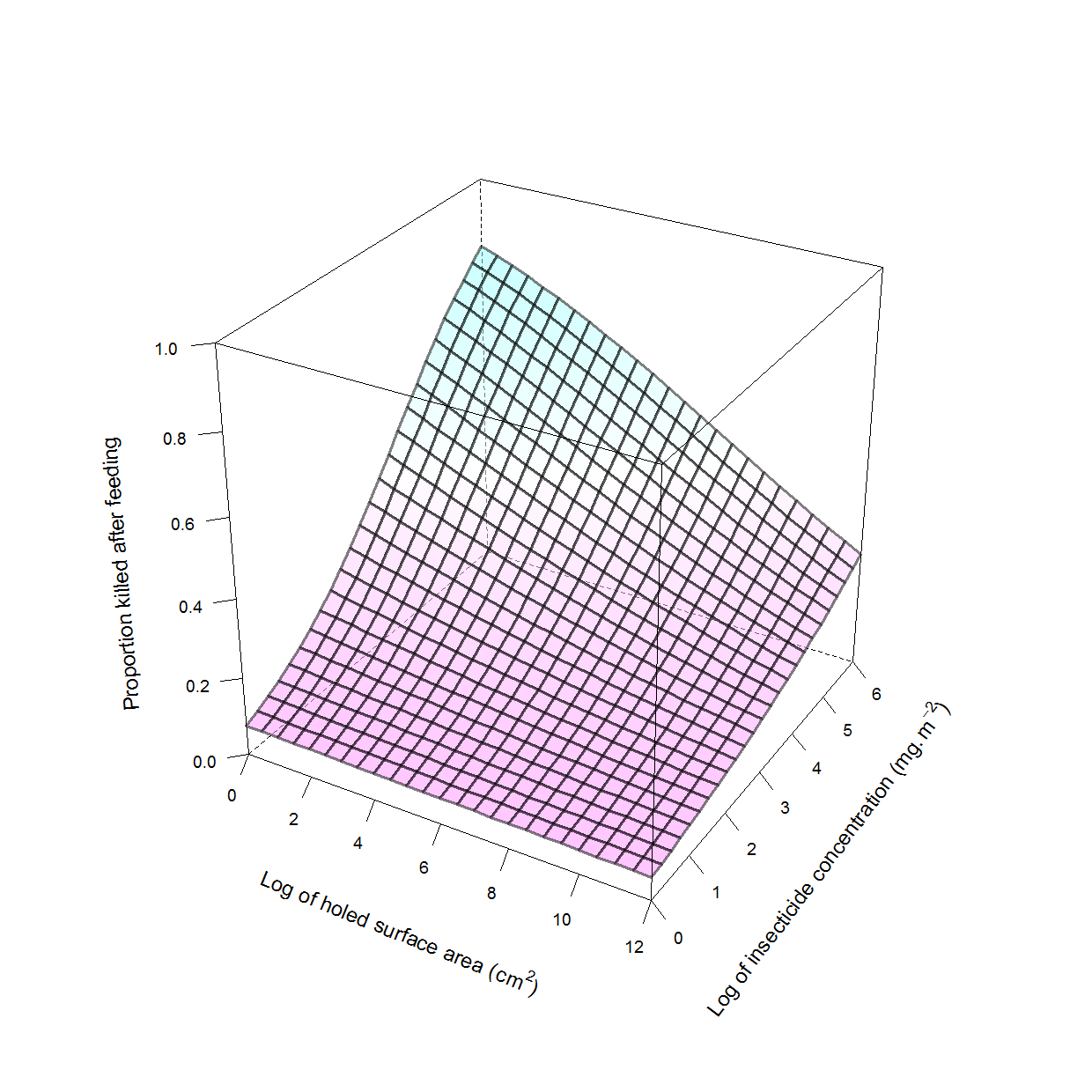

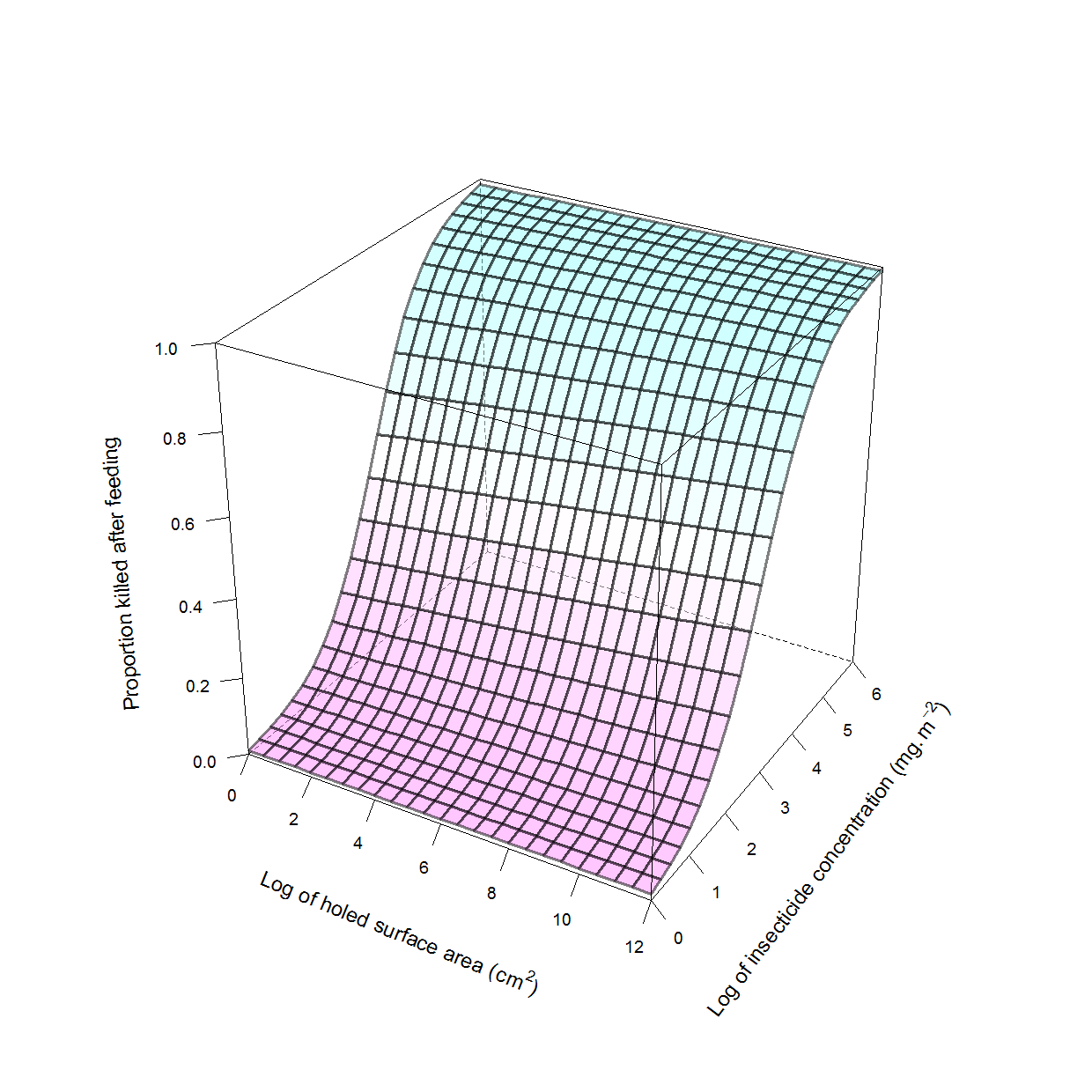


**Figure S4. The proportion of mosquitoes being killed after feeding depending on the insecticide concentration and holed surface area in net**

Left panel: *An. albimanus* with lambdacyhalothrin ITNs; right panel: *An. gambiae* s.l. with lambdacyhalothrin ITNs.


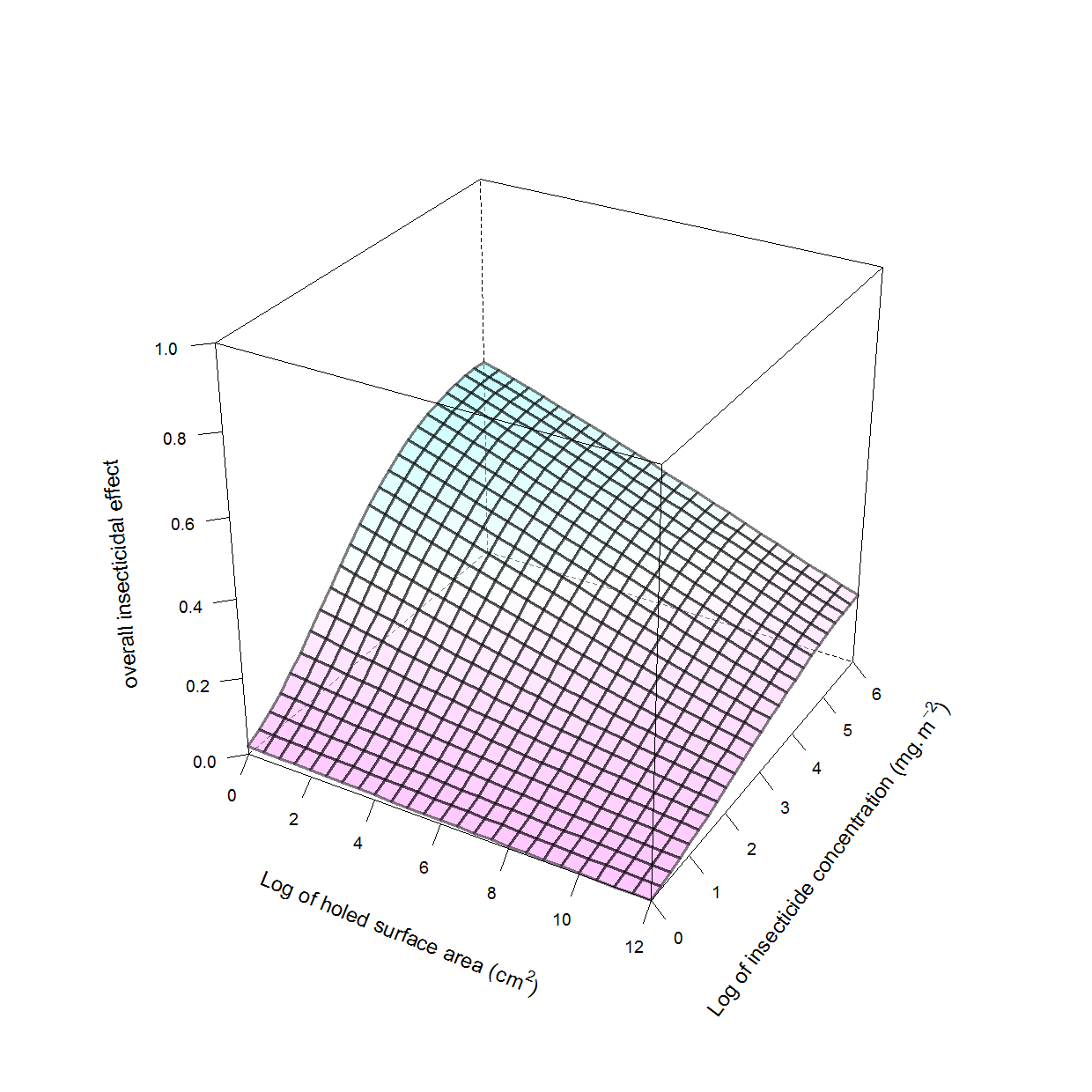

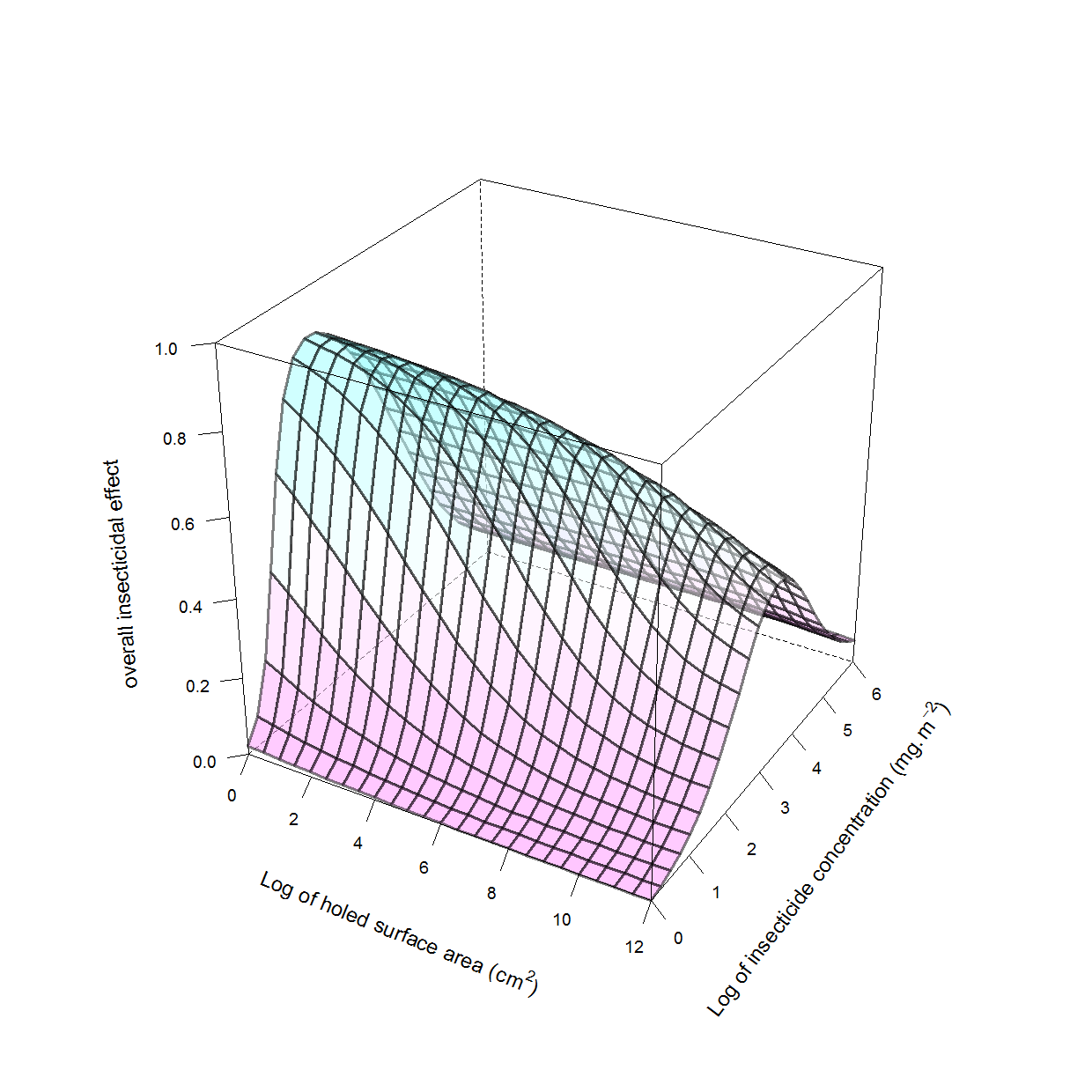


**Figure S5. The overall insecticidal effect on mosquitoes of a net depending on the insecticide concentration and holed surface area**

Left panel: *An. albimanus* with lambdacyhalothrin ITNs; right panel: *An. gambiae* s.l. with lambdacyhalothrin ITNs.


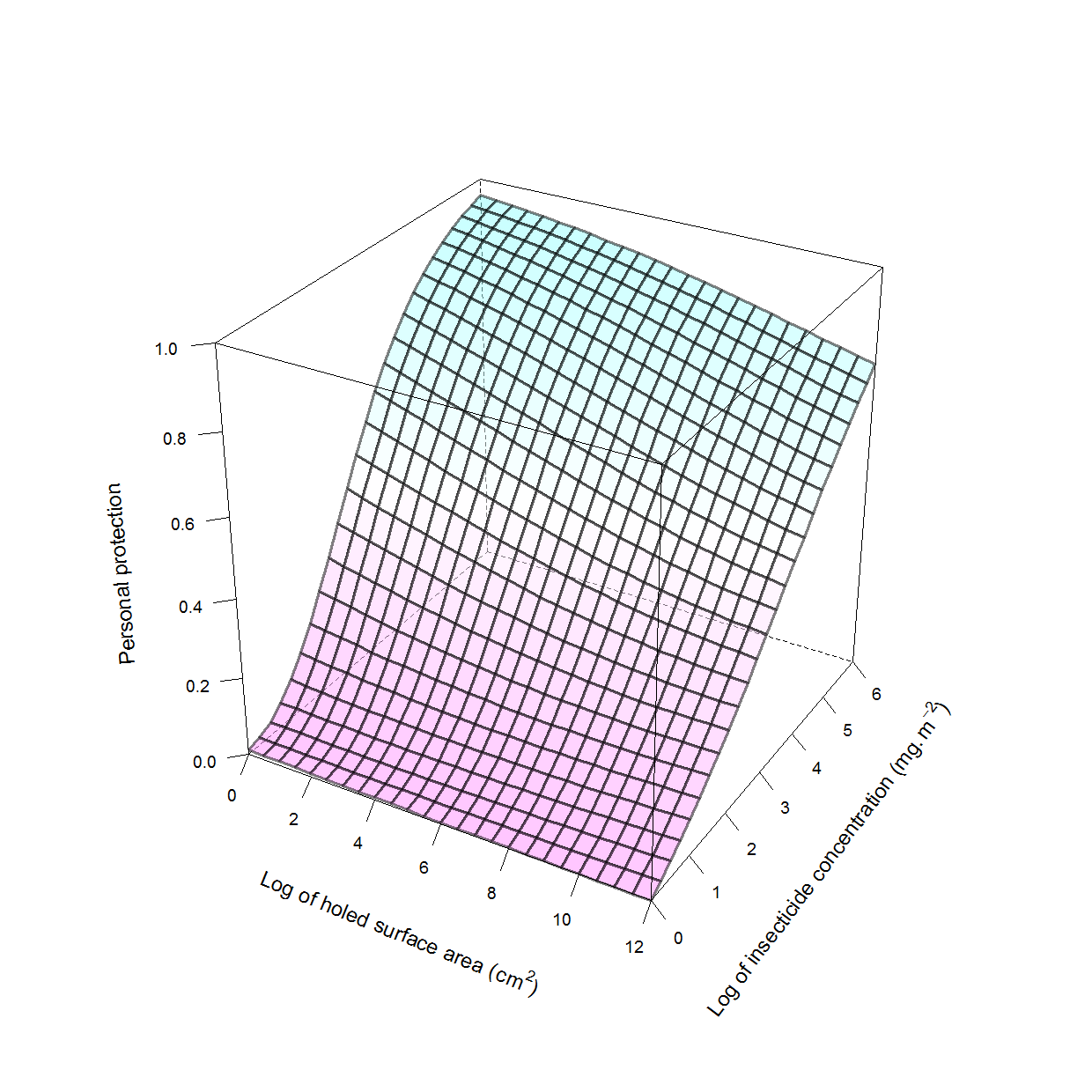

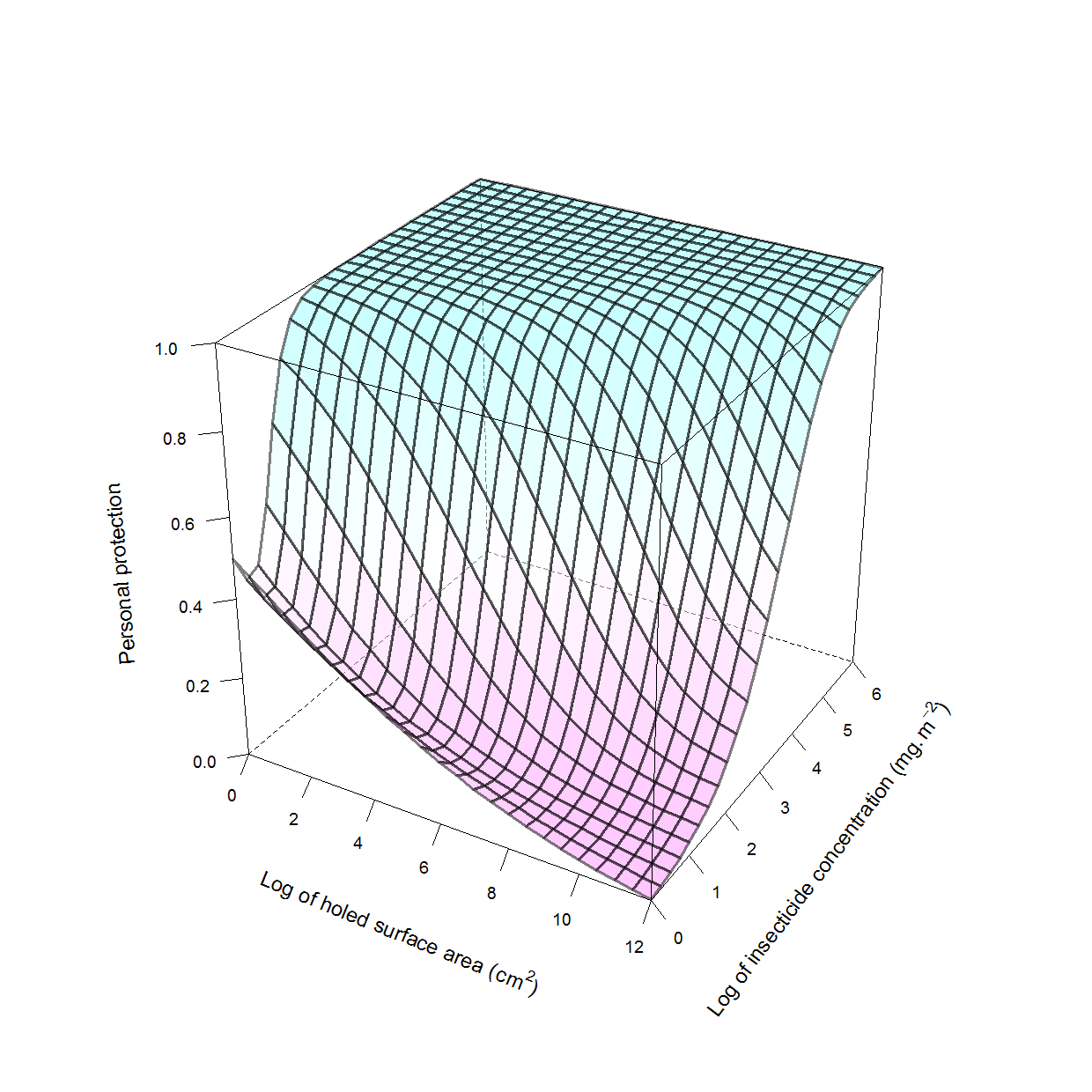


**Figure S6. The personal protection provided by a net against mosquito bites depending on the insecticide concentration and holed surface area**

Left panel: *An. albimanus* with lambdacyhalothrin ITNs; right panel: *An. gambiae* s.l. with lambdacyhalothrin ITNs.


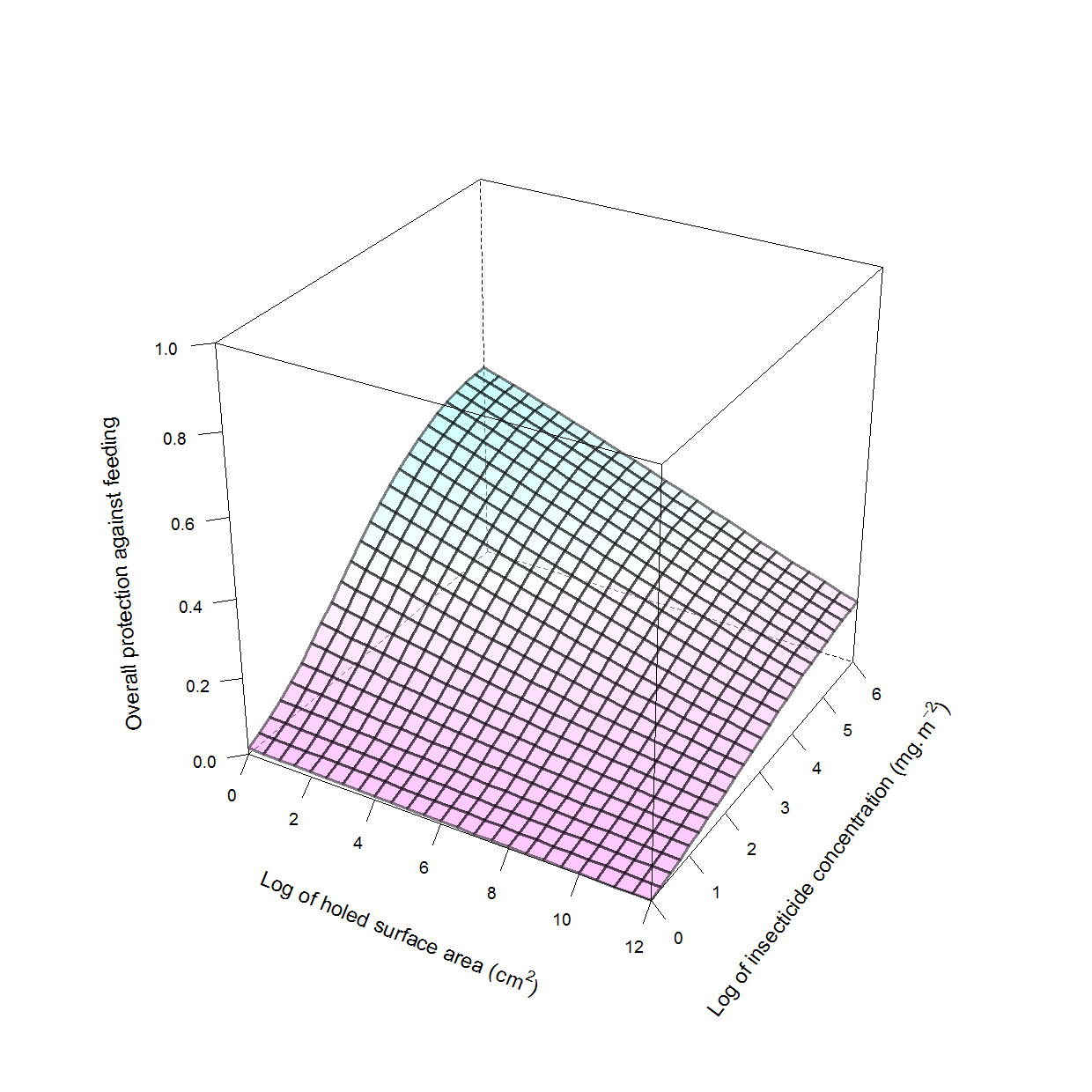

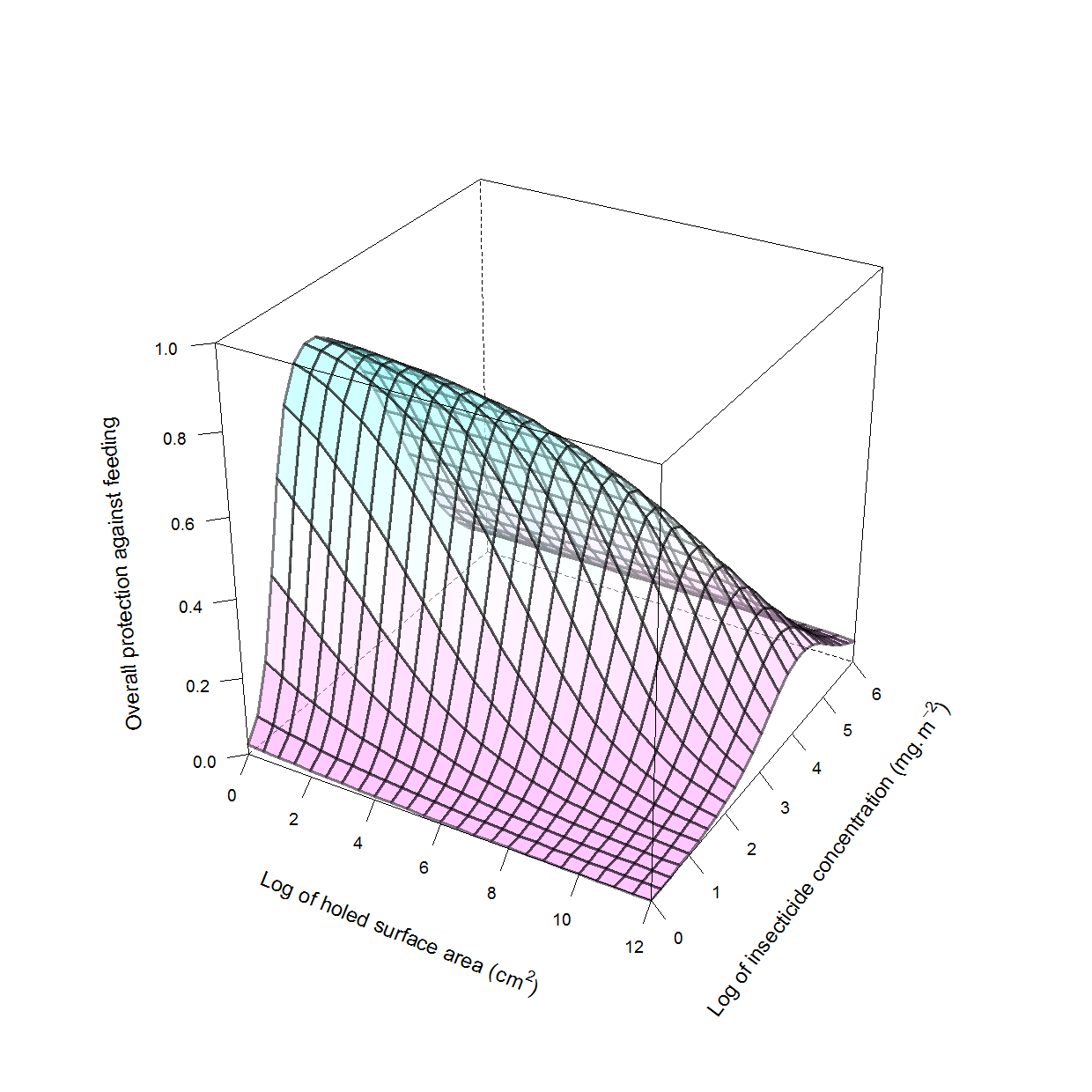


**Figure S7. The overall protection against feeding provided by a net depending on the insecticide concentration and holed surface area**

Left panel: *An. albimanus* with lambdacyhalothrin ITNs; right panel: *An. gambiae* s.l. with lambdacyhalothrin ITNs.

**
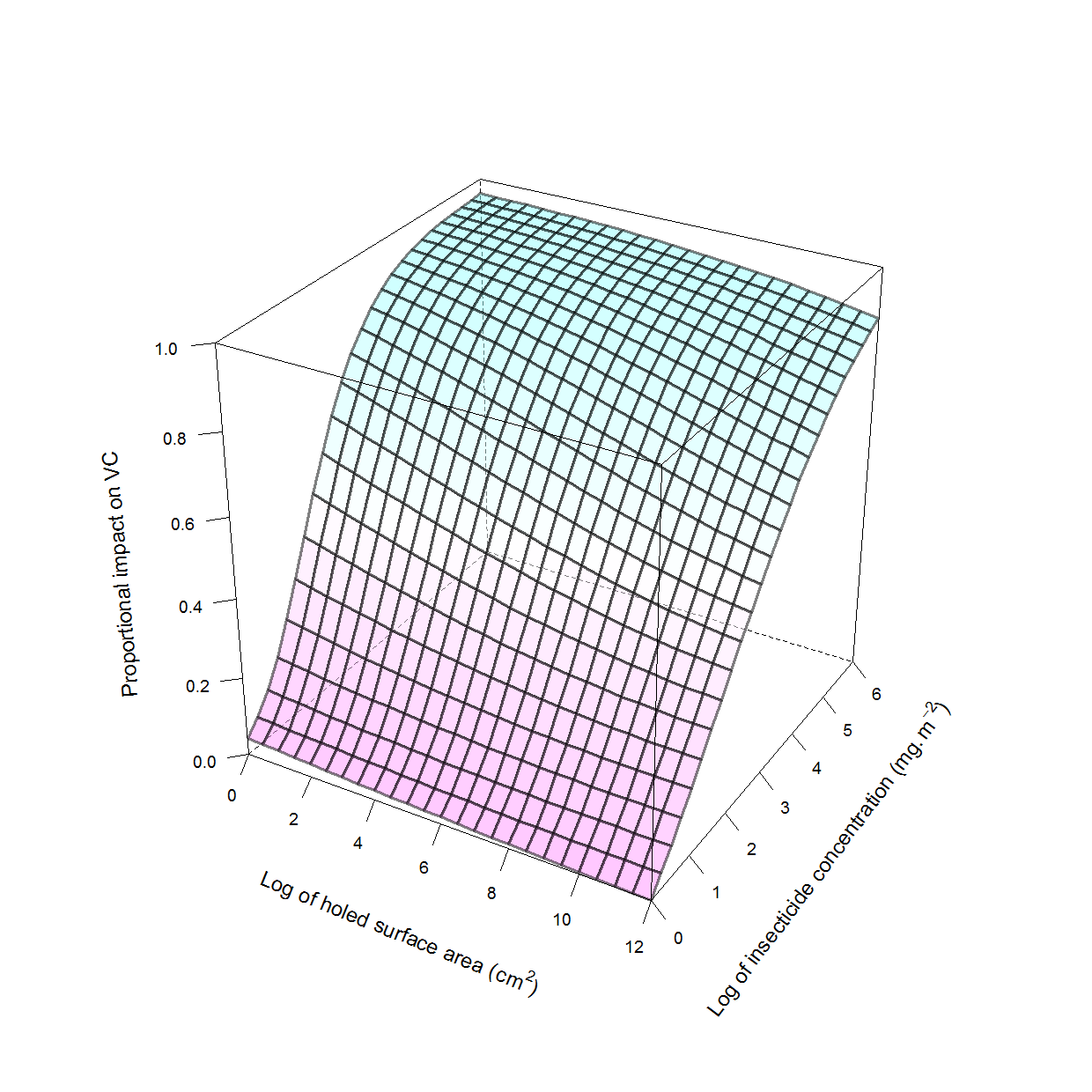

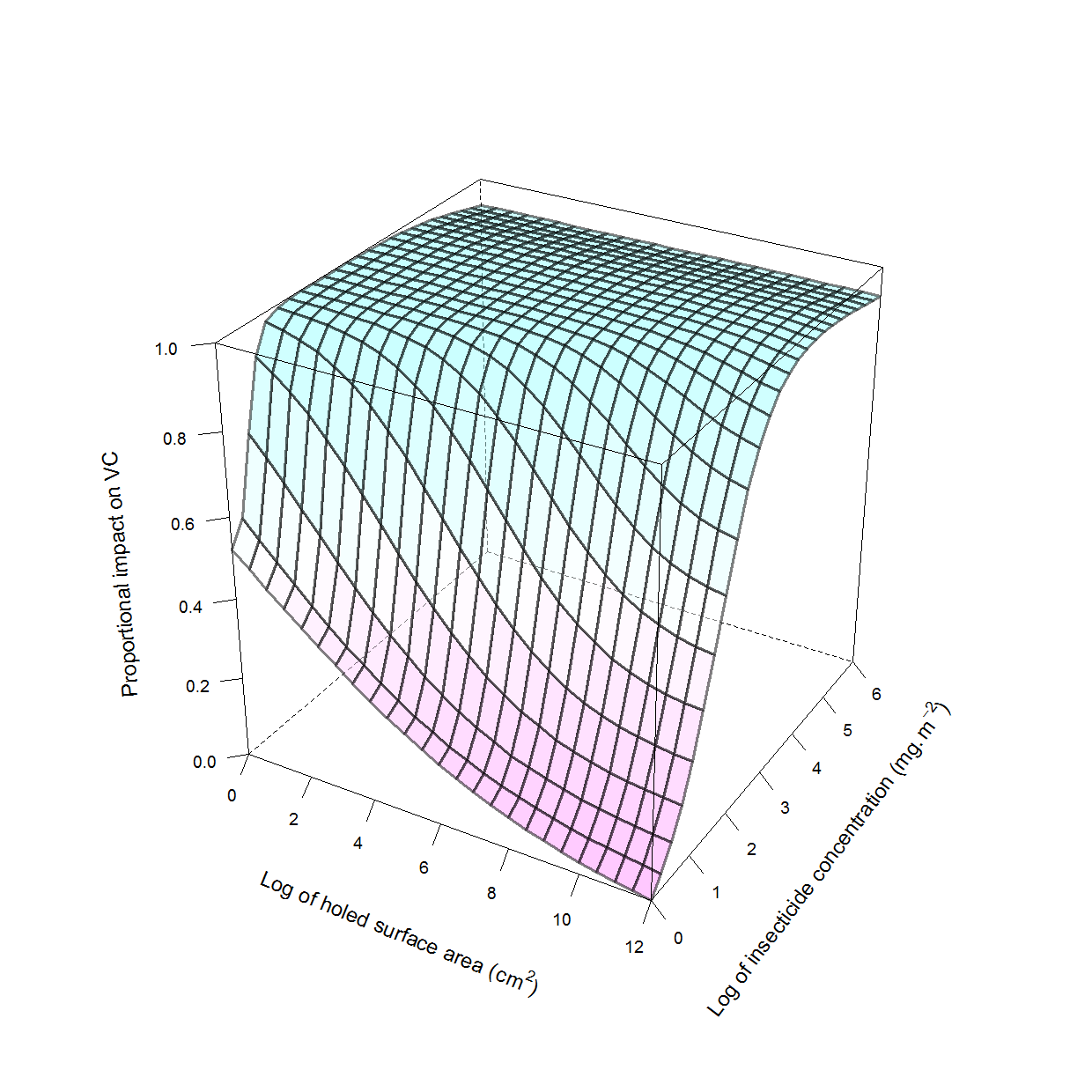
**

**Figure S8. The proportional impact on vectorial capacity of mosquitoes depending on the insecticide concentration and holed surface area in the nets in a population with a use coverage of 80% of identical nets, assuming all biting occurs when people are asleep**

Left panel: *An. albimanus* with lambda-cyhalothrin ITNs; right panel: *An. gambiae* s.l. with lambdacyhalothrin ITNs.

# Effects of IRS

## DDT IRS effects on *An. albimanus*

Indoors and outdoors of two experimental huts (one sprayed with 4% DDT 2 g/m^2^ and one control) in Belize, approximately 2 months after hut treatment until 15 weeks post-spray, Bangs (9) conducted human landing catches of *An. albimanus*. Outdoors 1,480 specimens were caught, while indoors in the treated hut only 73, and in the control 110 mosquitoes were caught. Thus, there was a reduction of biting of 33.63%. It is unknown how many entered or were killed preprandially, but the latter is likely to be very low, given the results from a concurrent mark-release-recapture experiment, where equal amounts of mosquitoes fed and died in treated and control huts. Less than 5% of mosquitoes were found dead on the floor, but for reasons that are unclear, the percentage mortality among exit box captures from treated and control huts after a 24-hour holding was over 50%. Therefore, it was assumed that there was no induced mortality by DDT. It was assumed that the reduction in availability to *An. albimanus* was a stable 33.63% over a 6 month period.

## DDT IRS effects on *An. gambiae*

Kuhlow (10) describes experimental hut studies with DDT in Natsini (12.150° N, 4.767° E) in north-west Nigeria (154 km from and at similar latitude as Malanville, the site used for the parameterization of lambdacyhalothrin ITNs and Bendiocarb IRS). Selected results are summarized in Table 5.

**Table S5: Experimental hut results with *An. gambiae* in Natsini grouped in four catagories, interpreted from data published by Kuhlow (10)**

| Treatment | Month post spray | Unfed alive | Unfed dead | Fed dead | Fed alive |
| --- | --- | --- | --- | --- | --- |
| Control |  |  |  |  |  |
|  | 0 | 646.0 | 33.1 | 23.8 | 5,477.1 |
|  | 1 | 2.8 | 0.5 | 0.0 | 105.7 |
|  | 2 | 2.0 | 0.4 | 0.0 | 75.7 |
|  | 3 | 4.4 | 0.7 | 1.5 | 56.4 |
|  | 4 | 31.1 | 1.0 | 0.8 | 269.1 |
|  | 5 | 212.5 | 7.8 | 0.8 | 1,776.8 |
| DDT 1.9 g/m^2^ |  |  |  |  |  |
|  | 0 | 590.2 | 407.6 | 2,496.9 | 590.2 |
|  | 1 | 13.3 | 7.6 | 30.7 | 13.3 |
|  | 2 | 4.9 | 2.6 | 12.6 | 4.9 |
|  | 3 | 7.0 | 0.7 | 8.0 | 14.2 |
|  | 4 | 57.2 | 3.3 | 16.3 | 116.1 |
|  | 5 | 298.9 | 25.4 | 212.9 | 606.9 |

For the simulations, effects at different time-interval mid points were linearly interpolated. Calculated effects over time are depicted in Figure 9 panel b. It was assumed that after 6.5 months, the effects were zero.

## Bendiocarb IRS effects on *An. albimanus*

In an experiments with whole netted existing houses, Bown and colleagues (11) found in Mexico with bendiocarb and deltamethrin that there was no deterrent effect, as the number of mosquitoes captured was generally higher in control houses than in treated houses. Mortality of those few mosquitoes found in the interior of the houses was high. There was some blood-feeding inhibition in the first month, and perhaps also the second month with bendiocarb. It was estimated that the bloodfeeding reduced 19% compared to the control after 7 weeks, 14% less after 11 weeks, and was the same as controls (0%) at 15 weeks. Mortality decreased with bendiocarb in months post treatment.

The plots of Bown and colleagues (11) show that bloodfeeding in the control hut varied strongly with time, ranging 43–70%. In some months, blood feeding was higher in treatment arms than in control for deltamethrin, but not for bendiocarb. Because mortality was high in treatment huts, this resulted in a much higher proportion of attacking mosquitoes (those ending up unfed dead, fed dead or fed and alive) than in controls, and this relative proportion attacking fluctuated with time. With deltamethrin, It was estimated that the bloodfeeding reduced 18% compared to the control after 7 weeks, 0% less after 11 weeks, and same as controls (0%) at 15 weeks.

Mortality was at first lower but then peaked before decreasing again. This was presunably due to avoidance from treated walls with delta at high concentrations. Interestingly, mortality was higher in the unfed mosquitoes than in the fed mosquitoes with both bendiocarb and deltamethrin (possibly unfed mosquitoes were generally weaker, or were the once that had rested on walls pre-feeding).

Despite not knowing the proportions remaining resting inside, based on the low number of mosquitoes counted inside, this was presumably low.

For the simulations, effects at different time-interval mid points were linearly interpolated. Effects of bendiocarb on *An. albimanus* over time are depicted in Figure 9 panel c.

## Bendiocarb IRS effects on *An. gambiae*

In Malanville, at the same site used for the parameterization of lambdacyhalothrin ITNs, Agossa and colleagues, (12) tested the effect of bendiocarb, fenitrothion, pirimiphos methyl and lambdacyalothrin in an experimental hut trial against a wild free entering *Anopheles gambiae* s.l. population. Selected results for bendiocarb are summarized in Table 6. Because in the hut with lambdacyalothrin (data not shown), unexpectedly, 14% more mosquitoes were found than in the control, bias of the number of mosquitoes entering due to hut location could not be excluded, and possible deterrence from entry was ignored in the parameterization of the effects for bendiocarb.

**Table S6: Experimental hut results with *An. gambiae* in Malanville grouped in four catagories, interpreted from data published by Agossa and colleagues** (12)

| Treatment | Month post spray | Unfed alive | Unfed dead | Fed dead | Fed alive |
| --- | --- | --- | --- | --- | --- |
| Control |  |  |  |  |  |
|  | 0.5 | 7.0 | 0.0 | 0.6 | 144.4 |
|  | 1.5 | 2.0 | 0.0 | 0.3 | 70.7 |
|  | 2.5 | 2.0 | 0.0 | 0.3 | 76.7 |
|  | 5.5 | 15.9 | 0.1 | 0.5 | 121.5 |
| Bendiocarb 0.4 g/m^2^ |  |  |  |  |  |
|  | 0.5 | 0.1 | 3.9 | 71.1 | 1.9 |
|  | 1.5 | 10.1 | 0.9 | 6.4 | 70.6 |
|  | 2.5 | 14.9 | 16.0 | 33.1 | 30.9 |
|  | 5.5 | 4.8 | 0.2 | 3.0 | 61.0 |

For the simulations, effects at different time-interval mid points were linearly interpolated. It was assumed that after 6.5 months, the effects were zero.

Effects over time are depicted in Figure 9 panel d.


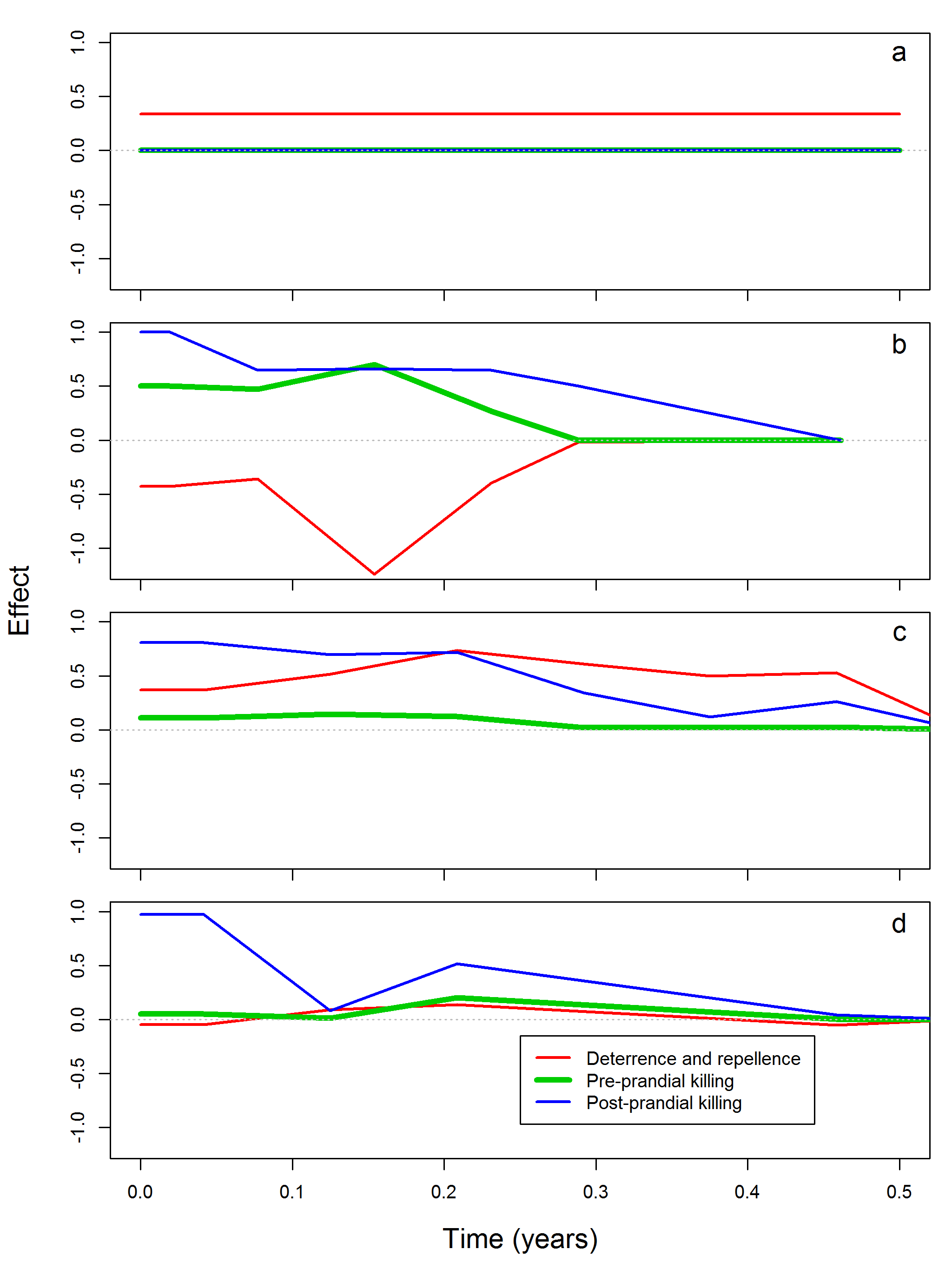


**Figure S9. Effect of indoor residual spray on mosquitoes**

**a** DDT on *An. albimanus*; **b** bendiocarb on *An. albimanus*; **c** DDT on *An. gambiae*; **d** bendiocarb on *An. gambiae*. The deterrence and repellence (from attacking a protected human host) can be negative, when more mosquitoes are dead and / or fed in intervention arms than in control arms. This occurred e.g. in the study of the effect of bendiocarb on *An. albimanus*, where only a small proportion of mosquitoes were fed in the control arm.

# References

1. Chitnis N, Smith T, Steketee R. A mathematical model for the dynamics of malaria in mosquitoes feeding on a heterogeneous host population. JBiolDyn. 2008;2(3):259-85.

2. Chitnis N, Smith T, Schapira A. Parameter values for transmission model. 2010. p. 17.

3. Briet OJ, Penny MA. Repeated mass distributions and continuous distribution of long-lasting insecticidal nets: modelling sustainability of health benefits from mosquito nets, depending on case management. Malar J. 2013;12:401.

4. Morgan J, Abilio AP, do Rosario Pondja M, Marrenjo D, Luciano J, Fernandes G, et al. Physical durability of two types of long-lasting insecticidal nets (LLINs) three years after a mass LLIN distribution campaign in Mozambique, 2008-2011. Am J Trop Med Hyg. 2015;92(2):286-93.

5. Arredondo-Jimenez JI, Rodriguez MH, Loyola EG, Bown DN. Behaviour of Anopheles albimanus in relation to pyrethroid-treated bednets. Med Vet Entomol. 1997;11(1):87-94.

6. Randriamaherijaona S, Briet OJ, Boyer S, Bouraima A, N'Guessan R, Rogier C, et al. Do holes in long-lasting insecticidal nets compromise their efficacy against pyrethroid resistant *Anopheles gambiae* and *Culex quinquefasciatus*? Results from a release-recapture study in experimental huts. Malar J. 2015;14:332.

7. N'Guessan R, Corbel V, Akogbeto M, Rowland M. Reduced efficacy of insecticide-treated nets and indoor residual spraying for malaria control in pyrethroid resistance area, Benin. EmergInfectDis. 2007;13(2):199-206.

8. Briet OJ, Smith TA, Chitnis N. Measurement of overall insecticidal effects in experimental hut trials. ParasitVectors. 2012;5:256.

9. Bangs MJ. The susceptibility and behavioral response of Anopheles albimanus Weidemann and Anopheles vestitipennis Dyar and Knab (Diptera: Culicidae) to insecticides in northern Belize, Central America. Ann Arbor: UMI; 1999.

10. Kuhlow F. Field experiments on the behavior of malaria vectors in an unsprayed hut and in a hut sprayed with DDT in Northern Nigeria. Bull World Health Organ. 1962;26:93-102.

11. Bown DN, Frederickson EC, Cabanas GA, Mendez JF. An evaluation of bendiocarb and deltametrin applications in the same Mexican village and their impact on populations of Anopheles albimanus. Bull Pan Am Health Organ. 1987;21(2):121-35.

12. Agossa FR, Aikpon R, Azondekon R, Govoetchan R, Padonnou GG, Oussou O, et al. Efficacy of various insecticides recommended for indoor residual spraying: pirimiphos methyl, potential alternative to bendiocarb for pyrethroid resistance management in Benin, West Africa. Trans R Soc Trop Med Hyg. 2014;108(2):84-91.
